# Progress in multi-omics studies of osteoarthritis

**DOI:** 10.1186/s40364-025-00732-y

**Published:** 2025-02-11

**Authors:** Yuanyuan Wei, He Qian, Xiaoyu Zhang, Jian Wang, Heguo Yan, Niqin Xiao, Sanjin Zeng, Bingbing Chen, Qianqian Yang, Hongting Lu, Jing Xie, Zhaohu Xie, Dongdong Qin, Zhaofu Li

**Affiliations:** 1 First Clinical Medical College, Yunnan University of Chinese Medicine, Kunming, Yunnan China; 2 School of Basic Medical Sciences, Yunnan University of Chinese Medicine, Kunming, Yunnan China; 3 Key Laboratory of Traditional Chinese Medicine for Prevention and Treatment of Neuropsychiatric Diseases, Yunnan University of Chinese Medicine, Kunming, Yunnan China

**Keywords:** Osteoarthritis, Omics, Genomics, Transcriptomics, Proteomics

## Abstract

Osteoarthritis (OA), a ubiquitous degenerative joint disorder, is marked by pain and disability, profoundly impacting patients' quality of life. As the population ages, the global prevalence of OA is escalating. Omics technologies have become instrumental in investigating complex diseases like OA, offering comprehensive insights into its pathogenesis and progression by uncovering disease-specific alterations across genomics, transcriptomics, proteomics, and metabolomics levels. In this review, we systematically analyzed and summarized the application and recent achievements of omics technologies in OA research by scouring relevant literature in databases such as PubMed. These studies have shed light on new potential therapeutic targets and biomarkers, charting fresh avenues for OA diagnosis and treatment. Furthermore, in our discussion, we highlighted the immense potential of spatial omics technologies in unraveling the molecular mechanisms of OA and in the development of novel therapeutic strategies, proposing future research directions and challenges. Collectively, this study encapsulates the pivotal advances in current OA research and prospects for future investigation, providing invaluable references for a deeper understanding and treatment of OA. This review aims to synthesize the recent progress of omics technologies in the realm of OA, aspiring to furnish theoretical foundations and research orientations for more profound studies of OA in the future.

## Introduction

Osteoarthritis (OA) is one of the most common chronic joint diseases, characterized by the gradual degeneration of joint cartilage, the formation of bone spurs at the joint margins, and joint inflammation, primarily affecting the quality of life of middle-aged and elderly individuals [1]. In 2020, an estimated 595 million individuals were afflicted with OA, representing 7.6% of the global population, with a 132.2% increase in the total number of cases since 1990 [2]. As a multifactorial disorder, the pathogenesis of OA involves mechanical stress, genetic predispositions, metabolic dysregulation, and biomechanical imbalances [3]. With disease progression, individuals may experience pain [4], stiffness, and functional impairment, substantially impacting the quality of life and imposing a significant burden on healthcare systems [5].

Although current treatment strategies, such as pharmacological management [6], physical therapy [7], and eventual joint replacement surgery [8], can alleviate symptoms to some extent, they do not halt the disease's progression. Therefore, a deeper understanding of the pathophysiological mechanisms of OA is crucial for the development of novel therapeutic approaches [9].

With advances in molecular biology and bioinformatics, omics technologies have become a powerful suite of tools for in-depth studies of human diseases [10]. These technologies, including genomics, transcriptomics, proteomics, metabolomics, and epigenetics, provide comprehensive biomolecular data that reveal the multilayered nature of OA pathophysiology. Particularly, in recent years, omics research has begun to unveil the molecular underpinnings of OA, offering new possibilities for early diagnosis, progression monitoring, and personalized treatment of the disease [9].

This review seeks to explore the latest advancements in omics research of OA, with a focus on how the integration of data across various omics layers can lead to a more comprehensive understanding of the pathophysiology of this complex disease. By examining the current literature, we will discuss the contribution of omics to unraveling the mechanisms underlying OA and how they may facilitate the development of new therapeutic strategies.

## Overview of the pathogenesis of OA

### Abnormal activation of the innate immune system

**Fig. 1:**
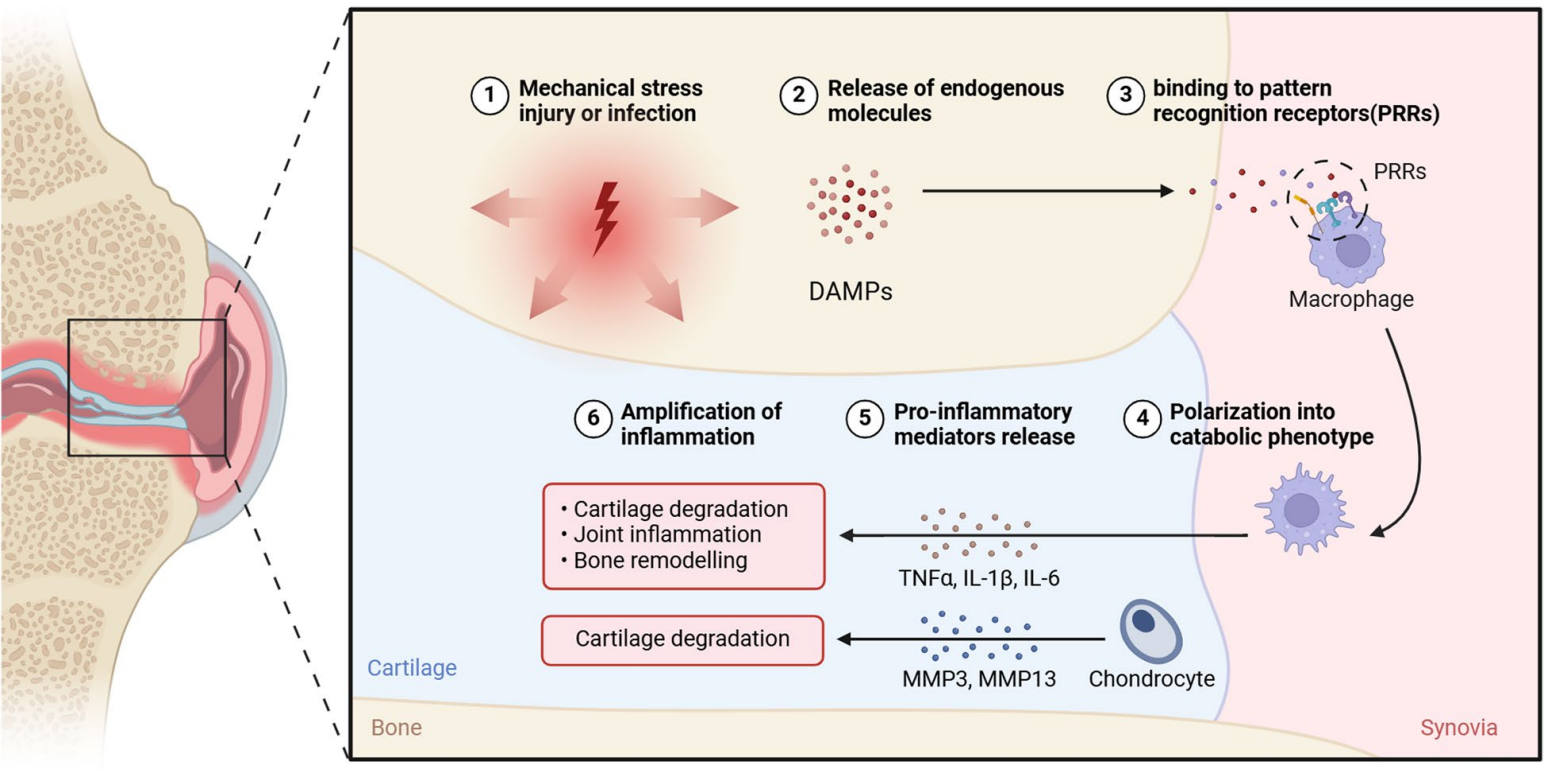
The Role of the Innate Immune System in the Pathogenesis of Osteoarthritis (OA). When joint cartilage or surrounding tissues are injured, endogenous molecules such as damage-associated molecular patterns (DAMPs), are released into the extracellular environment. DAMPs activate innate immune cells, such as macrophages, by binding to pattern recognition receptors (PRRs), thereby triggering a cascade of inflammatory reactions. This leads to the release of cytokines, chemokines, and other inflammatory mediators, resulting in further joint damage and exacerbation of inflammation. Activated immune cells produce pro-inflammatory cytokines, such as interleukin-1 beta (IL-1β), tumor necrosis factor-alpha (TNF-α), and interleukin-6 (IL-6), which are highly expressed in the synovium and promote inflammatory responses leading to cartilage degradation, joint inflammation, and bone remodeling. Concurrently, chondrocytes are also subjected to cartilage degradation under the influence of matrix metalloproteinases 3 (MMP3) and 13 (MMP13)

Chow et al. emphasized the involvement of inflammation in the development of OA [11]. In the pathogenesis of OA, the inflammatory response is primarily mediated by the innate immune system [12]. When joint cartilage or surrounding tissues are injured, endogenous molecules such as damage-associated molecular patterns (DAMPs), are released into the extracellular environment [13]. DAMPs activate innate immune cells, such as macrophages and dendritic cells, by binding to pattern recognition receptors (PRRs) like Toll-like receptors (TLRs), thereby triggering a cascade of inflammatory reactions. This leads to the release of cytokines, chemokines, and other inflammatory mediators, resulting in further joint damage and exacerbation of inflammation. Activated immune cells produce pro-inflammatory cytokines, such as Interleukin-1beta (IL-1β), Tumor necrosis factor alpha (TNF-α), and Interleukin-6 (IL-6), which are highly expressed in the synovium and promote inflammatory responses leading to cartilage degradation. If the inflammatory response persists, it may lead to chronic inflammation (Fig. 1). In OA, chronic low-grade inflammation can lead to further degeneration of joint cartilage, sclerosis of subchondral bone, and the formation of osteophytes, thereby aggravating joint damage and pain [14].

### Oxidative stress

Oxidative stress arises from an imbalance within the body's oxidative and antioxidant regulatory systems, leading to an overproduction of oxidative intermediates that exceed the normal antioxidant neutralization capacity, causing tissue and cellular damage. In OA, chondrocytes are subjected to oxidative stress, resulting in an imbalance between the intracellular oxidative and antioxidant systems. This imbalance is characterized by an excessive generation of reactive oxygen species (ROS), which can damage cellular lipids, proteins, and DNA, leading to cellular injury, apoptosis, and inflammation [15].

### Programmed cell death

#### Apoptosis

**Fig. 2:**
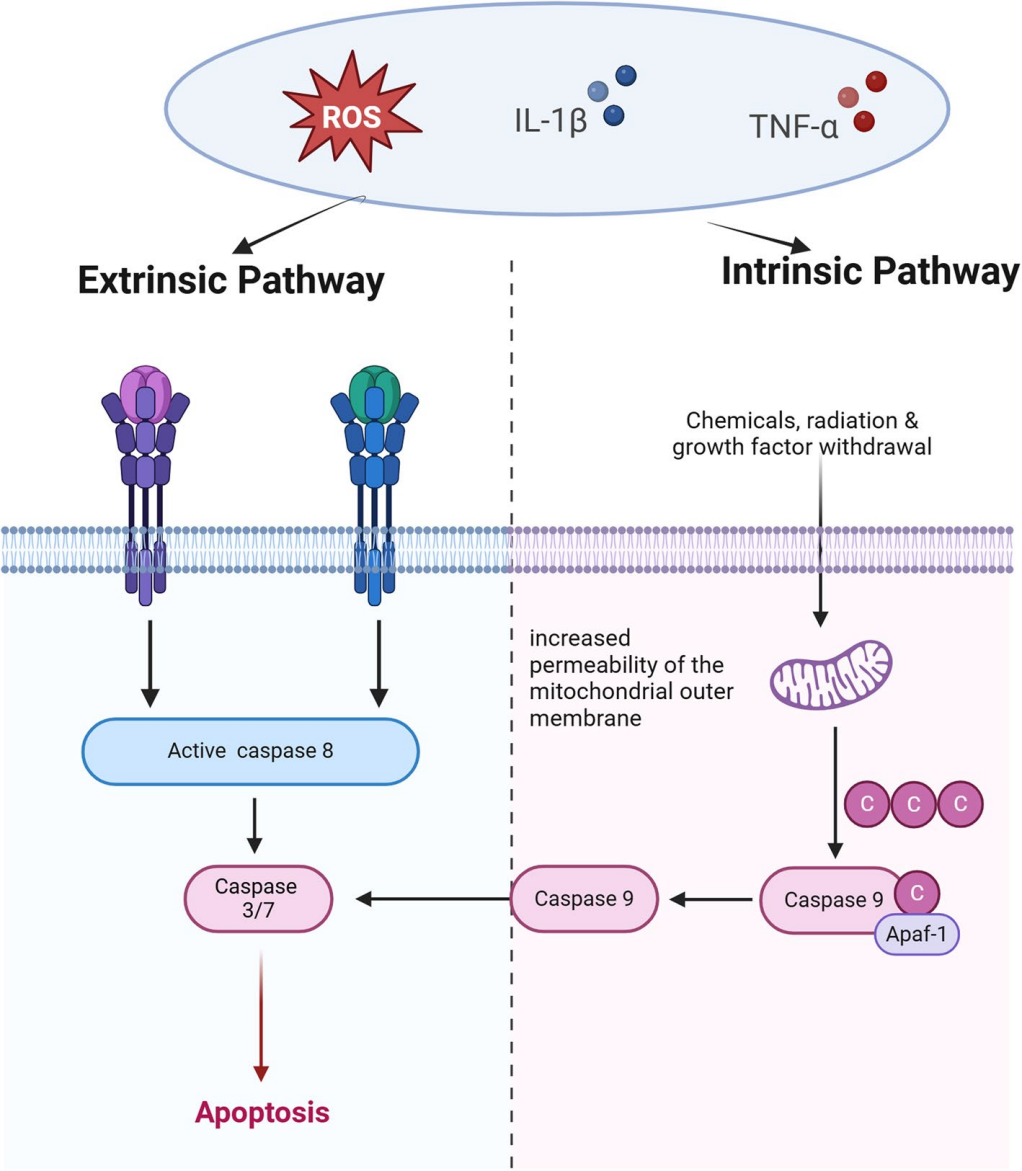
The process of apoptosis in OA. Chronic mechanical stress, pro-inflammatory cytokines such as IL-1β and TNF-α, and ROS can all initiate apoptosis in chondrocytes. Apoptosis primarily occurs through two pathways: the intrinsic pathway and the extrinsic pathway. The intrinsic pathway involves increasing the permeability of the mitochondrial outer membrane, releasing cytochrome c, which binds to Apaf-1 to form the apoptosome, activating caspase-9, and subsequently activating caspase-3/7, leading to cell apoptosis. The extrinsic pathway is mediated by death receptors (such as Fas and TNF receptors), which activate caspase-8, that in turn activates caspase-3/7, leading to cell apoptosis

Long-term mechanical stress, pro-inflammatory cytokines such as IL-1β and TNF-α, and oxidative stress products (such as ROS) are all inducers that can initiate chondrocyte apoptosis [12, 16]. Apoptosis primarily occurs through two pathways: the intrinsic and extrinsic routes. The intrinsic pathway involves increased permeability of the mitochondrial outer membrane, releasing cytochrome c, which binds to Apaf-1 to form an apoptosome, activating caspase-9, and subsequently activating caspase-3, leading to programmed cell death. The extrinsic pathway is mediated by death receptors (such as Fas and TNF receptors), activating caspase-8, which directly or indirectly activates caspase-3 (Fig. 2). Apoptotic chondrocytes reduce the production of cartilage matrix components (such as type II collagen and aggrecan), leading to cartilage damage. Released signaling molecules increase the expression of matrix metalloproteinases (MMPs), further promoting the degradation of the cartilage matrix [17].

#### Autophagy

**Fig. 3:**
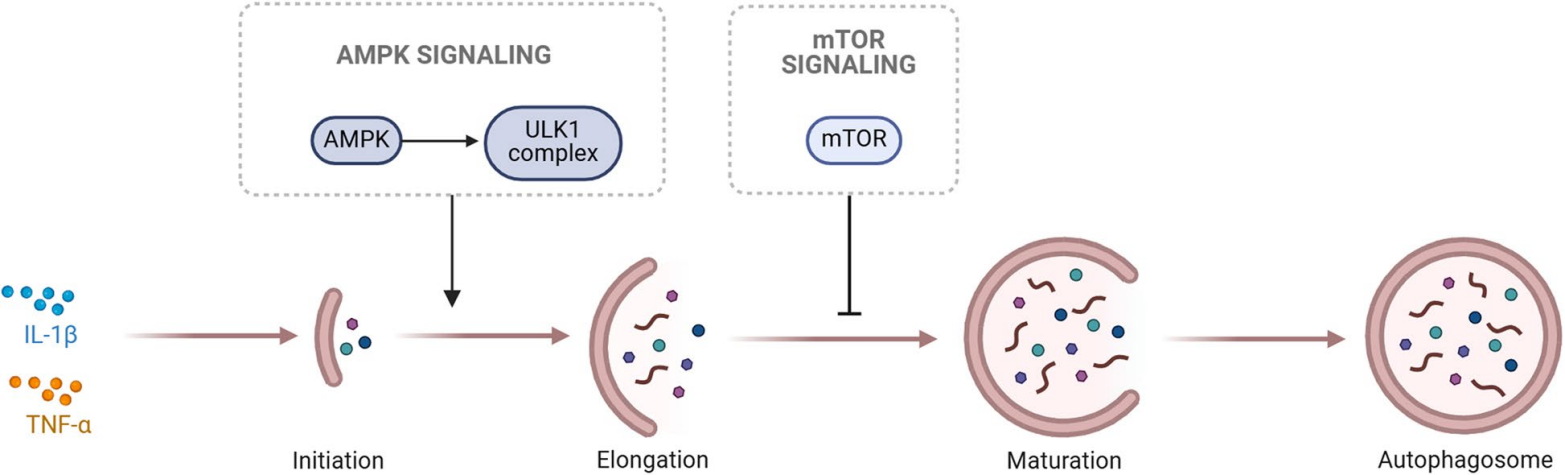
The process of pyroptosis in OA. Inflammatory factors such as IL-1β and TNF-α can activate autophagy to form autophagosomes. AMPK signaling pathways initiate autophagy by activating the ULK1 complex. mTOR signaling pathways can inhibit autophagy

Autophagy is an intracellular degradation and recycling process that is crucial for maintaining chondrocyte homeostasis. In OA, an imbalance in autophagy may lead to chondrocyte dysfunction, affecting the balance of cartilage matrix synthesis and degradation [18]. Inflammatory factors such as IL-1β and TNF-α can activate autophagy to form autophagosomes, which encapsulate damaged proteins and organelles within the cell and fuse with lysosomes to form autolysosomes, degrading and recycling these encapsulated materials. In chondrocytes, autophagy alleviates oxidative stress by providing nutrients and energy, clearing damaged mitochondria, and maintaining cell survival. Autophagy also regulates the balance of cartilage matrix metabolism, preventing matrix degradation. In synovial cells, autophagy helps maintain cellular function, regulates synovial fluid production and joint lubrication, and reduces inflammatory responses by degrading pro-inflammatory factors. Autophagy also regulates the function of osteoblasts and osteoclasts, participates in bone remodeling processes, maintains bone tissue homeostasis, and prevents osteoporosis and joint injury. Autophagy is primarily regulated through the mTOR and AMPK signaling pathways, with the former inhibiting autophagy and the latter initiating autophagy by activating the ULK1 complex (Fig. 3). Additionally, autophagy can delay or prevent apoptosis to a certain extent, protecting chondrocytes and synovial cells, but excessive autophagy may lead to autophagic cell death, further exacerbating the pathological process of OA.

#### Pyroptosis

**Fig. 4:**
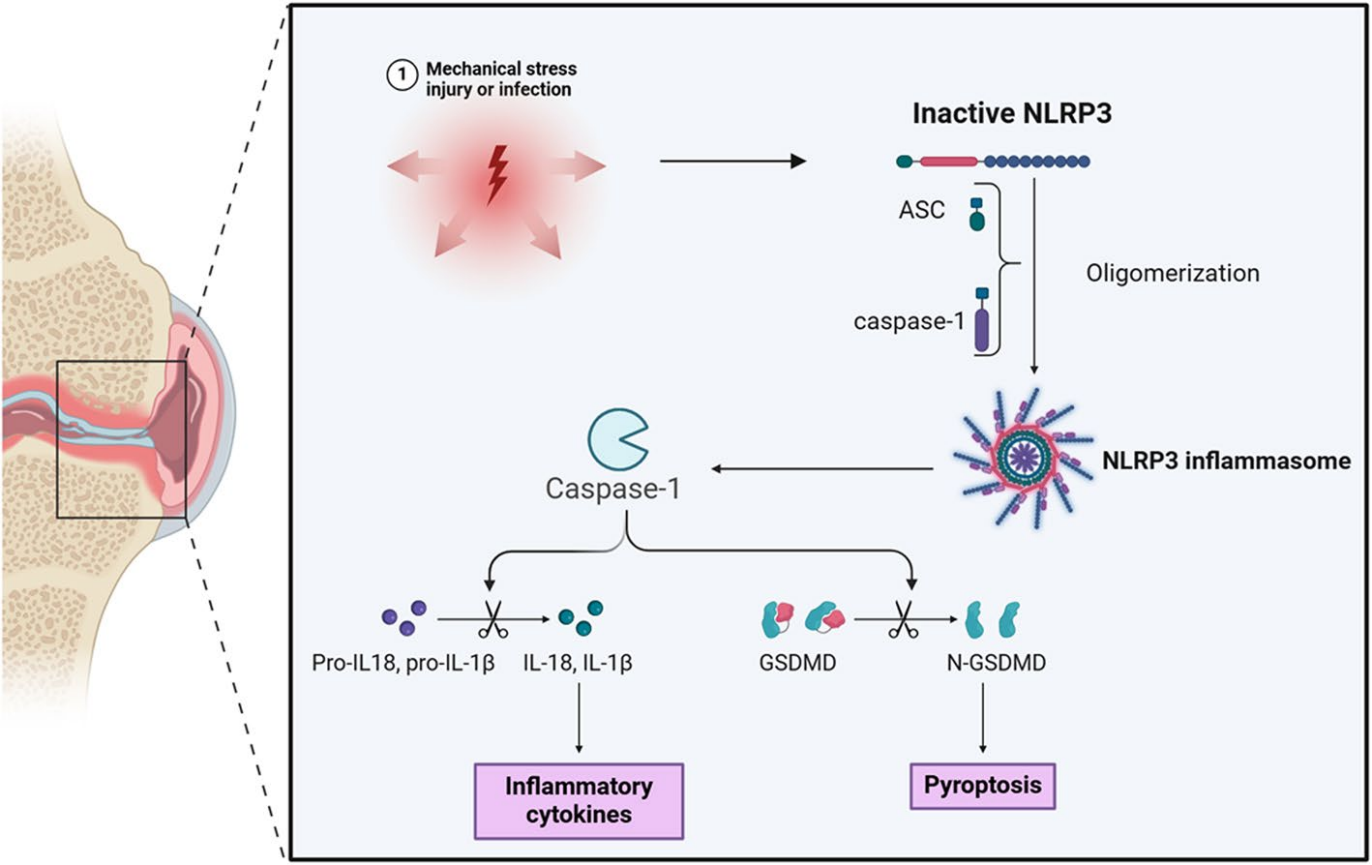
The process of pyroptosis in OA. Mechanical stress, injury and infection can activate the NLRP3 inflammasome. Once activated, the NLRP3 inflammasome recruits ASC (apoptosis-associated speck-like protein containing a pyrin domain), forming an inflammasome complex that promotes the recruitment and self-cleavage of procaspase-1, activating caspase-1. Activated caspase-1 cleaves the pro-inflammatory cytokine precursors pro-IL-1β and pro-IL-18, generating active IL-1β and IL-18. Additionally, caspase-1 cleaves gasdermin D (GSDMD), whose N-terminal fragment forms a pore-forming protein, leading to cell membrane rupture and the release of pro-inflammatory cytokines such as IL-1β and IL-18. These molecules activate adjacent cells and the immune system, triggering a strong inflammatory response that leads to pyroptosis

Pyroptosis is an inflammatory form of programmed cell death that plays a complex but important role in OA [19]. Initially, tissue damage, metabolites, and microbial products in OA can activate the NLRP3 inflammasome. Once activated, the NLRP3 inflammasome recruits ASC (apoptosis-associated speck-like protein containing a pyrin domain), forming an inflammasome complex that promotes the recruitment and self-cleavage of procaspase-1, activating caspase-1. Activated caspase-1 cleaves the pro-inflammatory cytokine precursors pro-IL-1β and pro-IL-18, generating active IL-1β and IL-18. Additionally, caspase-1 cleaves gasdermin D (GSDMD), whose N-terminal fragment forms a pore-forming protein, leading to cell membrane rupture and the release of pro-inflammatory cytokines such as IL-1β and IL-18, as well as DAMPs (Fig. 4). These molecules activate adjacent cells and the immune system, triggering a strong inflammatory response that leads to pyroptosis.

#### Ferroptosis

Ferroptosis in OA is caused by dysregulated iron metabolism leading to excessive iron accumulation, which generates a large amount of reactive oxygen species (ROS), triggering lipid peroxidation and antioxidant defense failure. Depletion of glutathione and inactivation of glutathione peroxidase 4 lead to the accumulation of lipid peroxides, damaging the cell membrane and inducing cell death [20]. The release of lipid peroxides and DAMPs from ferroptosis activates inflammatory responses, increasing the expression of inflammatory factors and MMPs, leading to the degradation of the cartilage matrix. This is an important mechanism for inducing or exacerbating inflammatory responses in OA.

In summary, the innate immune system, oxidative stress, and programmed cell death are pivotal in the pathogenesis of OA. These factors not only directly participate in the degradation of joint tissue but also influence cellular metabolism and functional status. The intricate molecular interactions and regulatory networks at this level provide crucial insights into the pathophysiology of OA. However, traditional, single-disciplinary research approaches are inadequate in fully unraveling this complexity, necessitating more profound and comprehensive investigative tools. Omics technologies offer a more holistic and multidimensional dataset, enabling a deeper understanding of the molecular underpinnings of OA. In the following section, we will delve into how omics research plays a significant role in this field, and the novel perspectives it brings to the diagnosis and treatment of OA.

## Genomics studies of OA

Genomics is a pivotal branch of modern biomedical research, which, by analyzing an organism's complete genetic information, uncovers the complex interplay between genes and diseases [21]. In the study of OA, the application of genomics has significantly enriched our understanding of the genetic basis of this complex disease, revealing numerous crucial genetic markers and biological pathways.

### Genome‑wide association studies (GWAS)

Genome-Wide Association Studies (GWAS) is a powerful research tool used to search for associations between specific genetic variations and diseases among different individuals [22]. In OA research, GWAS has successfully identified multiple susceptibility loci for the disease. Cindy G. Boer and colleagues conducted a GWAS meta-analysis encompassing 826,690 individuals of European and East Asian descent, assessing 11 distinct OA phenotypes, including those affecting the knee, hip, fingers, thumbs, and spine. The study identified a total of 100 independent and significantly associated risk single nucleotide variants (SNVs), with 60 of these SNVs correlating to more than one OA phenotype. Furthermore, 52 of the identified SNVs represent previously unknown genetic risk factors for the disease [23].

A study leveraged data from two large cohorts: the Million Veteran Program (MVP) and the UK Biobank, revealing genetic variations associated with 27 OA loci-encompassing 10 novel loci-by integrating findings across diverse ancestries. Among these 27 loci, 476 single nucleotide polymorphisms were significantly correlated with OA. These observations corroborate previous findings and provide evidence that certain OA-associated regions are consistent across different human populations [24].

WNT9A has been identified as a potential novel pathogenic gene that may be involved in the pathogenic mechanisms of OA. In addition, several previously identified OA genetic loci appear to confer risk for the development of OA across multiple joints: these include loci for TGFα, RUNX2, COL27A1, ASTN2, IL11, and GDF5 [25].

### Functional genomics

Functional genomics has emerged as a transformative approach in the field of OA research, offering profound insights into the molecular mechanisms underlying this complex disease. By integrating various genomic data types, including gene expression profiles, genetic variants, and epigenetic modifications, functional genomics is accelerating the discovery of genes that are not only associated with OA but also play a functional role in its pathogenesis. Norbert Bittner and colleagues conducted a comprehensive genome-wide chromatin conformation analysis (Hi-C) on primary chondrocytes harvested from eight patients with knee osteoarthritis (KOA), generating a detailed map that correlates chromosomal architecture with genomic sequence. This study led to the identification of two novel candidate effector genes, SPRY4 and PAPPA (pregnancy-associated plasma protein A), these genes may be involved in gene regulation through enhancer-promoter interactions [26].

Utilizing high-throughput screening and bioinformatics tools, numerous candidate genes associated with OA have been identified and selected, such as ANO3 [27], WWP2 [28], Pitx1, Bhlhe40, Sh3bp4, and Unk [29]. These findings not only reveal how specific genetic variations increase the risk of OA by affecting the biological functions of chondrocytes and the integrity of joint tissue but also point to new directions for research to further understand how these variations influence the development and progression of the disease.

### Genetic risk scores (GRS)

Polygenic risk score (PRS) analysis, a pivotal tool for forecasting disease susceptibility, has demonstrated significant potential in advancing clinical practice. However, the majority of assessments validating the accuracy of PRS have been concentrated within populations of European descent. In an effort to enhance the precision of OA PRS, some researchers have integrated data on age, body mass index, gender, local multi-ancestral PRS, and GWAS from Meta-analyses of Trans-ancestry GWAS to further refine the PRS [23, 30].

GRS can also help to elucidate gene-environment interactions in OA. For example, the influence of mechanical stress [31] or obesity on OA risk may be modified by an individual's genetic background, as reflected in their GRS.

In research, GRS can facilitate the discovery of new biological pathways involved in OA and the identification of novel therapeutic targets. For instance, Astragalus membranaceus can modulate the NRF2/HO-1 pathway through quercetin, thereby alleviating ferroptosis in chondrocytes and exerting a therapeutic effect on OA [32]. Among the newly identified targets, seven correspond to primary candidate targets in databases, which are CHST3, VDR, TNFSF11, IGF1R, NR3C1, CHRM2, and NOS3. These loci will target signaling pathways such as the intra-chondral pathway and skeletal development, offering new avenues for targeted therapy in the treatment of OA [23].

## Transcriptomics studies of OA

Transcriptomics is a discipline that provides insights into the state of gene expression by analyzing the collection of all RNA molecules within specific cells or tissues. It occupies a central role in modern biological research, particularly in the field of disease mechanism studies. In the study of OA, transcriptomic analysis has become an essential tool for understanding the changes in key genes and signaling pathways during joint degeneration and pathology.

### Association of differential gene expression analysis with joint degeneration

Differential gene expression analysis is a key technique in transcriptomic research, enabling the identification of genes that are upregulated or downregulated in the articular cartilage of healthy individuals compared to those with OA. These genes are often involved in multiple biological processes, including the metabolism of chondrocytes, the synthesis and degradation of the extracellular matrix (ECM), inflammatory responses, and apoptosis. Li et al. conducted a study using cytokine-treated tissue-engineered cartilage as an in vitro model of OA to perform transcriptomic analysis. This approach allowed for the identification of key gene expression changes associated with OA development, specifically in the catabolic aspect of inflammation induce OA [33]. Similarly, Bergen et al. investigated the transcriptome of regenerating zebrafish scales to identify genes relevant to human bone diseases, including OA [34]. The study identified differentially expressed genes enriched for pathways related to ECM, ossification, and cell adhesion, providing potential targets for further research. Rourke et al. focused on transcriptomic analyses in human and murine infrapatellar fat pads to identify common profibrotic changes in OA. This study shed light on the molecular alterations occurring in adipose tissue in the context of OA [35]. Additionally, Wu et al. and Tuerxun et al. explored the transcriptomic landscape of temporomandibular joint OA, highlighting the potential of noncoding RNAs as clinical biomarkers and providing insights into their functional roles in OA [36, 37]. Moreover, Hu et al. investigated dysregulated mechanotransduction and ECM pathways in a rat model of OA through transcriptomic analyses of joint tissues [38]. This study identified key pathways involved in OA development, further elucidating the molecular mechanisms underlying this disease.

Furthermore, Wang et al. investigated the transcriptomic profile of OA synovial macrophages, revealing a tolerized phenotype compounded by a weak corticosteroid response [39]. This finding may help explain the limited efficacy of corticosteroid treatments in improving long-term OA prognosis. Sharma et al. utilized microarray profiling and bioinformatic analyses to discern the differential expression of genes in OA patients [40]. They identified the upregulation of AMTN and DKK2, and the downregulation of MSLN, highlighting their critical roles in OA-related pathological mineralization and bone remodeling.

### The role of long non-coding RNA in OA

Long non-coding RNAs (lncRNAs) have been identified as key players in the pathogenesis of OA. Abbasifard et al. discuss the role and function of lncRNAs in OA, highlighting their potential as biomarkers and therapeutic targets for the prevention and treatment of the disease [41]. Wang et al. collate recent studies on the role of lncRNAs in the pathogenesis of OA, aiming to provide insights for the prevention, diagnosis, and treatment of the disease. [42].

Similarly, He et. al focus on the regulation of lncRNAs in cartilage injury associated with OA, emphasizing the importance of understanding the molecular mechanisms involved [43]. Dai et al. provide a comprehensive review of the differential expression of lncRNAs in OA, as well as the mechanisms by which lncRNAs regulate chondrocyte function and cartilage matrix metabolism [44]. Katsoula et al. conducted a large-scale study on lncRNA expression, isoform switching, and alternative splicing in OA, revealing novel genomic complexities associated with the disease [45]. In a network biology approach, Ali et al. propose data integration as a useful strategy for creating lncRNA networks in arthritis, focusing on chondrocytes in OA and fibroblast-like synoviocytes in rheumatoid arthritis [46]. Ghafouri-Fard et al. highlight the emerging role of non-coding RNAs, including lncRNAs, in OA pathology, emphasizing the need for a specific angle to provide meaningful insights into the disease process [47]. Chen et al. discuss the biological functions of exosomal non-coding RNAs in OA, highlighting their role in modulating gene expression at post-transcriptional and post-translational levels [48]. Wen et al. investigate the molecular mechanism of the lncRNA KLF3-AS1, derived from mesenchymal stem cell exosomes, in repressing autophagy and apoptosis of chondrocytes in OA [49]. Finally, Zhang et al. focus on a specific small non-coding RNA, piRNA hsa_piR_019949, which promotes chondrocyte anabolic metabolism by inhibiting the expression of the lncRNA NEAT1 in chondrocytes [50].

### Regulatory role of microRNA and its disease relevance

MicroRNAs (miRNAs) are a class of short non-coding RNA molecules that regulate gene expression by degrading target mRNAs or inhibiting their translation.

Studies have shown differential expression of miRNAs in normal human articular cartilage compared to OA cartilage, indicating their potential role in disease progression [51]. Modulating miRNAs in the joint has been demonstrated to reduce OA in animal models, suggesting a novel therapeutic strategy for the management of OA [51]. Specific miRNAs have been implicated in the progression of OA. For example, miRNA-128A has been shown to interrupt circadian rhythmicity signaling, accelerating the progress of OA [52]. Additionally, miRNA-144 has been identified as having increased expression in both the early phase and end-stage of OA, highlighting its potential as a therapeutic target [53]. Furthermore, miRNA-140-3p has been associated with extensive downregulation of immune gene expression in an in vitro model of OA, further emphasizing the role of miRNAs in OA pathogenesis [54].

Recent research has also focused on the role of circulating miRNAs in OA. A distinct signature of circulating miRNAs has been identified in early radiographic knee OA, suggesting their potential as biomarkers for early disease detection [55]. Moreover, miRNAs have been implicated in muscle regeneration and diseases related to muscle dysfunction, such as atrophy, cachexia, osteoporosis, and OA, underscoring their diverse roles in musculoskeletal health [56]. Nanoparticles containing miRNA-224-5p have shown promise in balancing homeostasis, inhibiting cartilage degeneration, and alleviating synovial inflammation in OA, highlighting the therapeutic potential of miRNA-based interventions [57]. Further research is needed to elucidate the precise mechanisms by which miRNAs contribute to OA progression and to translate these findings into clinical applications for improved OA management.

Overall, transcriptomic analyses have provided valuable insights into the molecular mechanisms underlying OA pathogenesis, highlighting the potential for targeted therapeutic interventions based on the identified pathways and genes. Further research in this area is essential to advance our understanding of OA and develop effective treatments for this debilitating condition.

## Proteomics studies of OA

Proteomic studies of OA have made significant advancements in unraveling the molecular mechanisms of the disease and in the discovery of potential biomarkers.

### Application of proteomics in articular cartilage

Dudek et al. explored the daily dynamics in cartilage physiology using circadian time series proteomics [58]. By analyzing the proteomic changes in cartilage over time, this study provides insights into the temporal regulation of proteins involved in OA progression. Furthermore, Wang et. al conducted a differential proteomic analysis of tibial subchondral bone in guinea pigs with spontaneous OA to investigate molecular alterations in early OA [59]. Their findings identified significant differences in protein expression between male and female guinea pigs, highlighting potential sex-specific mechanisms in OA development. Park et al. focused on proteomic analysis of meniscus cartilage in OA patients [60]. By characterizing the proteomic profiles of meniscal tissue and developing an assay for ECM proteins, this study contributes to understanding the molecular changes in OA-affected cartilage. In patients with OA, differentially expressed proteins are implicated in the dysregulation of complement and coagulation cascades. A significant increase in SerpinA5 within the subchondral bone of OA patients has been observed, suggesting that the disruption of coagulation and complement pathways plays a role in the progression of OA and may provide a potential therapeutic target for the disease [61].

### The role of proteomics in identifying inflammatory markers

Giordano et al. conducted a study to assess serum levels of inflammatory markers in patients with KOA using Proximity Extension Array technology [62]. Their findings aimed to evaluate the associations between these markers and clinical pain intensity in KOA patients. This study highlights the importance of proteomic analysis in identifying potential biomarkers for OA. Urinary proteomics, through the identification of peptide biomarkers, has demonstrated the dysregulation of collagen synthesis and inflammation, and has also highlighted novel inflammatory markers for the identification and differentiation of distinct subtypes of inflammatory arthritis [63]. Quantitative proteomics was used to reveal the protective effects of a compound called EDS against OA by attenuating inflammation and modulating immune responses [64].

### Proteins analysis in synovial membrane

Researchers have identified proteins that are differentially expressed in synovial membrane, linking menopause with the progression of OA and potentially leading to novel diagnostic and therapeutic strategies [65].Elevated levels of vitronectin fragments (amino acids 381–397) have been identified in the serum of OA patients, which can interact with αVβ6 on human fibroblast-like synoviocytes to inhibit the activation of TGF-β1, potentially promoting fibrosis in OA [66]. A study has meticulously analyzed the metabolic, proteomic, and functional characteristics of THY1 + fibroblast-like synoviocytes in OA patients, uncovering an elevated expression of pyruvate dehydrogenase kinase 3 as a distinctive feature of proliferative THY1 + FLS in these individuals and reveals how metabolic changes in synovial fibroblasts contribute to OA [67]. Lastly, Wijesinghe et.al highlight the significance of obesity in both load-bearing and non-load bearing joints in changing the inflammatory molecular endotype OA synovial fibroblasts, providing a rational for the therapeutic targeting of specific synovial fibroblasts subsets in specific patient populations with arthritic conditions [68].

### Proteomic analysis in synovial fluid

The protein composition in synovial fluid reflects the state of the intra-articular environment, and proteomic analysis can identify biomarkers associated with OA in synovial fluid. Synovial fluid proteomics has unveiled a plethora of biomarkers associated with arthritis, encompassing components of the complement system, inflammatory mediators, and markers of cartilage metabolism [69]. For instance, in patients with OA, there is an elevated presence of complement components such as C5, interleukin-6, and Cartilage Oligomeric Matrix Protein in synovial fluid.

Proteomic biomarkers of meniscal disease have been identified from synovial fluid samples derived from control knees as well as knees exhibiting varying severities of meniscal injury and OA [70]. The differential expression of 13 proteins was observed, suggesting a potential association with the progression of meniscal damage and OA. These findings may pave the way for the exploration of synovial fluid biomarkers in meniscal pathology.

Overall, these studies demonstrate the diverse applications of proteomics in understanding the pathogenesis, progression, and potential therapeutic targets for OA. By utilizing advanced proteomic technologies, researchers can uncover novel insights into the complex molecular mechanisms underlying OA.

## Metabolomics studies of OA

### Metabolic profiling of articular tissues

Recent studies have focused on understanding the metabolic pathways involved in OA pathogenesis, particularly in relation to cartilage health. Rocha et al. highlighted the impact of UDP-glucuronic acid and UDP-GlcNAc synthesis pathways on chondrogenic differentiation in OA cells, potentially affecting the production of cartilage ECM components. This emphasizes the importance of metabolic pathways in maintaining cartilage integrity [71]. Anderson et al. conducted an ex-vivo equine cartilage explant OA model study, identifying differentially abundant metabolites, proteins, and ECM-derived neopeptides associated with OA pathogenesis [72]. Similarly, Southan et al. investigated the metabolic signature of articular cartilage post-injury using an integrative analysis of transcriptomics and metabolomics, shedding light on the metabolic changes in cartilage tissue following mechanical injury [73]. Furthermore, Welhaven et al. conducted studies comparing metabolomic profiles of healthy and OA human cartilage, revealing distinct metabolic differences between the two conditions [74]. This comparative analysis highlighted specific metabolic pathways with unique regulation patterns in OA cartilage. Additionally, metabolomic methodologies have the capacity to elucidate the underlying mechanisms of osteophyte formation in OA, revealing metabolic alterations associated with this pathological development, including changes in amino acids, sulfonic acids, glycerophospholipids, and fatty acyls [75]. These metabolites are implicated in physiological and pathological processes such as cartilage dissolution, disruption of boundary layers, and the triggering of self-repair mechanisms. Notably, phenylalanine metabolism is significantly correlated with this degenerative process.

### Metabolic profiling of plasma

Research has uncovered numerous metabolites and metabolic pathways linked to OA [76]. Specific metabolites, such as lysophospholipids, phospholipids, arginine, branched-chain amino acids, and histidine, have been recognized as potential biomarkers for OA, highlighting amino acid metabolism, glycolysis, tricarboxylic acid cycle, and lipid metabolism as therapeutic targets [76, 77].

A systematic review of 32 population-based metabolomic studies utilizing plasma/serum, synovial fluid, cartilage, or subchondral bone samples has uncovered metabolic pathways implicated in OA, including energy metabolism, arginine and proline metabolism, taurine and hypotaurine metabolism, and glycerophospholipid metabolism [78].

Additionally, a study by Peevenage et al. focused on utilizing metabolomics to identify potential biomarkers and perturbed metabolic pathways in OA, emphasizing the need for personalized approaches to disease management [79]. Serum metabolomics has shown promise in distinguishing individuals with OA from healthy controls. Analysis of serum metabolomics in rats with OA revealed significant differences in metabolite levels, including decreased estradiol2 levels in the OA group [80]. Therefore, Anterior cruciate ligament damage could significantly disrupt energy metabolism [81]. Dysregulated glucose levels may impair chondrocyte function, promoting OA. Metabolomic studies using plasma/serum have identified altered metabolic profiles in patients with OA, highlighting the potential of metabolomics in understanding the pathophysiology of the disease [82].

### Metabolic profiling of synovial fluid

Several studies have utilized metabolomic analysis of synovial fluid to identify potential biomarkers and perturbed metabolic pathways in OA. Clarke et al. employed Nuclear Magnetic Resonance spectroscopy to analyze biofluids for OA, highlighting the importance of metabolomic profiling in OA research [83]. Hahn et al. investigated the effects of long-term exercise and a high-fat diet on synovial fluid metabolomics and joint structural phenotypes in mice, emphasizing that obesity strengthened synovial fluid metabolite links to blood glucose and inflammation [84]. Wallace et al. compared metabolites in synovial fluid and serum to identify potential biomarkers of joint injury in a mouse model of posttraumatic OA, shedding light on the pathophysiology of the disease [85]. Damyanovich et al. focused on 1H NMR metabolic profiling of synovial fluid from patients with anterior cruciate ligament tears and hemarthrosis, highlighting the diagnostic potential of metabolomic analysis in OA [86]. Laus et al. demonstrated that the synovial fluid metabolome can differentiate between healthy joints and OA-affected joints in horses, showcasing the utility of metabolomics in disease diagnosis [87]. Furthermore, Arjun et. al conducted a systematic review to analyze metabolites and metabolic pathways involved in OA pathogenesis and early treatment [76]. These studies collectively underscore the importance of metabolomics in identifying biomarkers, perturbed pathways, and potential therapeutic targets in OA.

### Metabolic profiling of synovial membrane

Cai et al. utilized bioinformatics analysis to identify key biomarkers and immune infiltration in the synovial membrane of OA [88]. By constructing a protein–protein interaction network and performing module analysis, they aimed to uncover important factors contributing to OA development. In a similar vein, Murillo-Saich et al. conducted metabolomic profiling of synovial membrane to reveal biomarkers of synovial inflammation in OA patients [89]. Their study shed light on the metabolic alterations associated with OA pathogenesis, providing valuable information for the development of targeted therapeutic strategies.

### Metabolic profiling of urine

Jiang et al. detected the urine of rats with OA via gas chromatography—time-of-flight/mass spectrometry (GC-TOF/MS), and discovered that the metabonomic pathogenesis of OA might be associated with disorders in amino acid metabolism, energy metabolism, fatty acid metabolism, vitamin B6 metabolism and nucleic acid metabolism [90]. Furthermore, urinary metabolomics has unveiled shared metabolic pathways and biomarkers between OA and other conditions, such as perturbation in glutamine metabolism, aiding in the enhanced comprehension of OA's pathological mechanisms and offering potential targets for the development of novel therapeutic strategies [91].

Overall, metabolomics plays a crucial role in understanding the metabolic changes associated with OA, offering valuable insights into disease mechanisms, potential biomarkers, and therapeutic interventions.

## Epigenetics studies of OA

Recent research in the field of OA has highlighted the interplay between genetics and epigenetics in the development and progression of the disease [92]. Genome-wide scans have identified numerous risk loci associated with OA, while epigenetic studies have focused on DNA methylation, histone modifications, and regulatory RNAs in joint tissues [92, 93].

### The role of DNA methylation in OA

Recent studies have delved into the epigenetic mechanisms underlying OA pathogenesis, particularly focusing on DNA methylation patterns in peripheral blood mononuclear cells as potential predictors of radiographic progression in OA patients [94]. Additionally, the interplay between OA and body mass index has been explored in relation to leptin promoter methylation in Taiwanese adults [95]. Enhancers have also been a subject of interest in OA research, with a genome-wide analysis revealing aberrant DNA methylation patterns in enhancers in knee and hip OA patients [96]. Furthermore, the genetic and epigenetic regulation of COLGALT2 has been investigated, demonstrating its association with OA genetic risk [97]. A systematic review has highlighted the importance of DNA methylation in bone metabolism-related diseases such as osteoporosis and OA, emphasizing the need for further investigations in this area [98]. Moreover, the methylation status of the CAMP gene promoter has been linked to chondrocyte apoptosis in OA patients, shedding light on the role of epigenetic modifications in OA pathophysiology [99]. Epigenome-wide studies have provided a comprehensive view of OA in primary tissues, offering insights into the epigenetic landscape of the disease [100]. Machine learning models based on peripheral blood DNA methylation have been developed to predict knee OA progression, addressing the need for accurate biomarkers in OA clinical research [101]. Histone demethylases have also been implicated in OA pathogenesis, with inhibition of KDM7A/B histone demethylases showing protective effects against OA by restoring H3K79 methylation [102]. Additionally, epigenomic profiling of the infrapatellar fat pad in OA patients has revealed genome-wide DNA methylation profiles, providing valuable insights into the epigenetic regulation of OA [103]. The study reveals that matrix stiffness modulates H3K27me3 demethylation by the opening of mitochondrial permeability transition pores and the translocation of plant homeodomain finger protein 8, subsequently affecting the expression of Mmp13 and Bax genes, which exacerbates the progression of OA [104].

### The impact of histone modifications on OA

Histone modifications play a crucial role in the pathogenesis of OA by regulating gene expression and cellular functions. Epigenetic mechanisms, such as DNA methylation and histone modifications, have been shown to impact the progression of OA by influencing chondrocyte fate and cartilage homeostasis [105]. The dysregulation of histone modifications, including histone methylation and acetylation, can lead to aberrant gene expression patterns in OA, contributing to disease development and progression [106]. Histone H3K9 methylation has been implicated in temporomandibular joint OA, with decreased levels observed in degenerated condylar articular cartilage in aged mice [107]. By analyzing human and murine models, genetic variations in the DOT1L gene were found to correlate with OA and stature, and the histone-modifying enzyme DOT1L-mediated H3K79 methylation was linked to the Wnt signaling pathway, offering a novel therapeutic target for epigenetic-based treatments of OA [108].

The study found that OA chondrocytes increase IL-1β and IL-8 production, which are epigenetically regulated, and that these cytokines stimulate matrix metalloproteinases and aggrecanase synthesis, leading to ECM degradation. Thus, examining histone post-translational modifications is essential for understanding the epigenetic control of inflammatory factors in OA [109]. In the context of OA, understanding the epigenetic players and chromatin marks involved in articular cartilage is essential for defining epigenetic therapies [110]. By focusing on DNA methylation and histone modifications, researchers aim to elucidate how these epigenetic marks influence the pathogenesis of OA and potentially lead to novel diagnostic and therapeutic strategies. Overall, unlocking the potential of histone modification in regulating bone metabolism holds promise for advancing our understanding of OA and developing targeted interventions [111].

## Single-cell omics studies of OA

### Innovations and applications of single-cell RNA sequencing technology

Single-cell transcriptomics has emerged as a powerful tool in understanding the molecular landscape of various diseases, including OA. Recent studies have utilized single-cell RNA sequencing (scRNA-seq) to uncover the heterogeneity and molecular changes associated with OA [112]. For instance, Sebastian et al. conducted a study on mouse articular chondrocytes and identified nine chondrocyte subtypes with distinct molecular profiles, highlighting injury-induced early molecular changes in response to joint trauma [113]. Similarly, Sun et al. compared chondrocyte states in femoral cartilage between healthy individuals and OA patients, revealing differences in cell subtypes and proposing a novel classification of each subtype [114]. Moreover, the immune landscape in post-traumatic osteoarthritis (PTOA) has been explored using single-cell transcriptomics. Sebastian et al. identified multiple immune cell types in the joint, such as neutrophils, monocytes, macrophages, B cells, T cells, NK cells, and dendritic cells, shedding light on PTOA-associated changes in the immune microenvironment [115]. Additionally, Fan et al. integrated multi-omics data to unveil inflammatory and prehypertrophic cell populations as key contributors to knee cartilage degeneration in OA, defining 11 chondrocyte populations, including pre-inflammatory and inflammatory chondrocyte populations [116]. Furthermore, single-cell transcriptomics has been instrumental in studying other aspects of OA, such as the trajectories of cartilage stem/progenitor cells (CSPCs) in disease progression [117] and the cellular composition and mechanisms in subchondral bone marrow lesions of KOA [118]. These studies have provided new insights into the molecular mechanisms underlying OA pathogenesis and potential therapeutic strategies. Additionally, through single-cell RNA sequencing of the infrapatellar fat pad and synovium, and the creation of a comprehensive single-cell atlas, it was discovered that APOE exerts a deleterious impact on cartilage [119]. In conclusion, single-cell transcriptomics has revolutionized our understanding of OA by uncovering cellular heterogeneity, molecular changes, immune responses, and key cell populations associated with disease progression. By analyzing the transcriptomic profiles at a single-cell resolution, researchers have been able to identify novel cell subtypes, characterize disease-specific markers, and elucidate the complex interplay of different cell types in the pathogenesis of OA.

### Applications of single-cell epigenomics

Studies have revealed alterations of DNA methylation and histone modifications in OA chondrocytes that may affect gene expression and thus participate in the pathogenesis of OA [120]. Single-cell analyses have been utilized to identify specific cell types contributing to the progression of KOA, such as anterior cruciate ligament fibroblasts [121]. These analyses have also been instrumental in understanding the cellular origins of complex diseases like type 1 diabetes and identifying new therapeutic targets for OA [122]. Additionally, the epigenome plays a crucial role in determining gene transcription and cell fate, as evidenced by the sculpting of the epigenome with histone modifications and transcription factor occupancy [123]. It was found that six RNA modification-related genes (ADAMDEC1, IGHM, OGN, TNFRSF11B, SCARA3 and PTN) were identified as possible biomarkers of OA and RA pathogenesis [124].

### Breakthroughs in single-cell proteomics

Single-cell proteomics technologies, especially mass spectrometry, face challenges in sensitivity and throughput but have made progress in revealing cell surface markers and intracellular signaling molecules related to OA. These technologies not only help understand cellular states in OA but also provide information for the identification of potential therapeutic targets. Inflammatory arthritis, including OA, has been studied using deep single-cell proteomics analysis to identify insufficient PD-1 expression during active autoimmune responses [125]. This analysis provides valuable information on the cellular biogeography of human bone marrow niches in OA, highlighting joint fold enrichment of all cell type pairs in the given neighborhood [126].

Overall, the integration of single-cell epigenomics with genetics and genomics holds promise for unraveling the complexities of OA and other related diseases, paving the way for targeted therapeutic interventions and personalized medicine approaches.

## Microbiomics studies of OA

In recent years, with the rapid development of microbiomics, researchers have begun to focus on the role of the microbiome, particularly the gut microbiome, in the development of OA. Microbiomics, the scientific study of microbial communities and their interactions with the host, has gradually revealed that gut microbes may affect joint health through various mechanisms in OA research.

### The association between gut microbiota and OA

Wei et al. conducted a study on the Xiangya Osteoarthritis Study participants to examine the association between gut microbiome and symptomatic hand osteoarthritis [127]. Their findings suggest a potential link between gut microbiota and the presence of symptomatic hand OA. Similarly, Yu et al. explored the causal role of gut microbiota in the development of OA, further supporting the idea that gut microbiota may play a role in the pathogenesis of OA [128]. In a study by Loeser et al., the association of increased serum lipopolysaccharide with obesity-related OA was investigated. While microbial dysbiosis was not found to be associated with OA, the presence of lipopolysaccharide in the blood was linked to obesity-related OA. This suggests a potential mechanism through which gut microbiota may influence the development of OA [129]. Ramires et al. also explored the association between gut microbiota and OA, raising the question of whether the disease may begin in the gut. Their study adds to the growing body of evidence supporting a connection between gut microbiota and OA [130]. Additionally, Wei et al. examined the association between gut microbiota and elevated serum urate levels, suggesting that microbiota dysbiosis may modulate these levels, potentially impacting OA development [131]. Furthermore, Xiang et al. conducted a systematic review on the association of low-grade inflammation caused by gut microbiota disturbances with KOA. Their review aimed to provide scientific evidence for this association, highlighting the role of gut microbiota in inflammatory processes related to OA [132]. Jiang et al. investigated the relationship between gut microbiota dysbiosis and hand synovitis prevalence, suggesting a potential role of bile acids as mediators in this association [133]. Lastly, Luo et al. conducted a comprehensive Mendelian randomization study to explore the causal link between gut microbiota, neurophysiological states, and bone diseases [134]. Their findings suggest that alterations in gut microbiota can impact cognitive ability, insomnia, and potentially reduce the risk of site-specific fractures and OA. Overall, these studies provide valuable insights into the potential association between gut microbiota and OA, highlighting the need for further research in this area.

### The role of oral-gut microbiome

In addition to gut microbiota, oral microbiota is also associated with the development of OA. Studies have suggested a dysbiotic association between the oral-gut microbiome and the pathogenesis of arthritis and prosthetic joint infections [135]. The gut microbiome has been identified as a significant contributor to musculoskeletal health and disease, with recent findings indicating that oral microbiota may also play a role in these conditions [136]. Research has proposed a possible relationship between rheumatoid arthritis (RA) and the microbiome of the oral cavity and gut, although this connection has not been systematically studied [137]. Changes in the composition of the oral-gut microbiota have been observed in patients with RA following treatment with methotrexate and non-surgical periodontal interventions [138]. Furthermore, perturbations in the oral microbiota have been linked to a higher risk for developing rheumatoid arthritis, suggesting that oral microbial dysbiosis may increase susceptibility to this condition [139]. The study of the oral microbiome in systemic diseases, including rheumatoid arthritis, has shown that both the oral and gut microbiomes are perturbed in these conditions [140]. Overall, more research is needed to fully understand the role of the oral-gut microbiome axis in OA and related diseases. Studies evaluating the impact of periodontal disease on quality of life and the use of oral supplements in managing musculoskeletal conditions highlight the potential importance of this area of research [141].

### The interaction between microbiota and cartilage

Recent research has highlighted the potential role of the gut microbiome in the pathogenesis of OA, suggesting an interaction between microbiomics and cartilage health [141]. By analyzing cartilage samples from human knees and hips, it was found that compared to healthy controls, OA samples exhibited reduced microbial diversity, and there was an increase in functions related to lipopolysaccharide production, phosphatidylinositol signaling, and nitrogen metabolism, while the function of sphingolipid metabolism was decreased [142]. This suggests that the microbiome within OA cartilage may influence the pathogenesis of OA by affecting inflammatory and metabolic processes. Similarly, Berthelot et al. identified gut microbial DNA signatures in human cartilage, suggesting that gut microbiota may reach the joints through the bloodstream, influencing the pathogenesis of OA [143]. The article introduces the concept of the "Cartilage-gut-microbiome axis," emphasizing the interaction between the gut microbiome and cartilage, which could provide new therapeutic targets for OA. The study found that the gut microbiome may directly affect cartilage health through the circulatory system. Dietary adjustments, probiotics and prebiotics use, and fecal microbiota transplantation might exert therapeutic effects on OA by inhibiting cartilage catabolism or apoptosis [144–147].

In summary, microbiomics has shown tremendous potential in OA research, not only helping us to understand the complex etiology of OA more deeply but also providing new directions for the development of new prevention and treatment strategies.

## Lipidomics studies of OA

### Lipidomics plays a role in the pathogenesis of OA

Kimmerling et al. investigated the role of fatty acid composition and metabolic inflammation in post-traumatic OA, suggesting that systemic factors may contribute to OA pathogenesis [148]. Rocha et al. identified a distinct lipidomic profile in the OA synovial membrane using mass spectrometry imaging, highlighting the importance of lipid composition in OA pathology [149]. Pousinis et al. explored alterations in plasma lipidomic profiles in a mouse model of OA, aiming to identify biomarkers of pain and pathology [150]. Casati et al. focused on bioactive lipid families in an in vitro model of OA, demonstrating the involvement of specific lipids in the inflammatory response [151]. Furthermore, Coras et al. proposed combining lipidomics with other omics techniques to enhance the characterization of cell types involved in arthritis pathogenesis, emphasizing the importance of comprehensive analysis of synovial membrane for a better understanding of OA [152]. Additionally, metabolic abnormalities, including lipid deposition in articular cartilage, have been linked to OA risk, highlighting the importance of lipidomic profiles in predicting obesity-associated OA [153].

### Application of lipidomics in diagnosis

Loef et al. investigated the association of lipid profiles with OA severity in knee and hand joints, suggesting a potential link between lipid composition and disease progression [154]. Furthermore, Li et al. identified diagnostic lipidomic biomarkers for seropositive and seronegative rheumatoid arthritis, highlighting the potential of lipidomics in distinguishing different arthritic conditions [155]. Tu et al. conducted a study that delved into the proteomic and lipidomic landscape of the infrapatellar fat pad in KOA, uncovering distinct molecular characteristics that could serve as potential diagnostic and therapeutic targets [156]. Eveque-Mourroux et al. investigated the differences in lipidomics and proteomics of cartilage between OA patients with and without type 2 diabetes, it was found that abnormal omega oxidation and fatty acid biosynthesis pathway may lead to imbalance of lipid metabolism [157].

### Application of lipidomics in prognosis assessment of OA

By examining the lipidomic profiles in plasma and synovial fluid, researchers have been able to predict obesity-related OA, synovitis, and wound healing, suggesting that lipidomic signatures may play a role in the early diagnosis and prognosis of OA [158]. The use of lipidomics to predict the response to prednisolone treatment in patients with inflammatory hand osteoarthritis indicates that lipidomic features may aid in the implementation of personalized medicine [159]. Targeted lipidomics has revealed the activation of resolution pathways in human KOA, offering potential therapeutic targets for OA treatment [160].

In the context of mesenchymal stem cell biology, bioactive lipids such as polyunsaturated fatty acid (PUFA) derived mediators (commonly known as eicosanoids), endocannabinoids (eCBs), and lysophospholipids (LPLs), and their roles in mesenchymal cells have been investigated [161]. This article provides a comprehensive overview of bioactive lipids in mesenchymal stem cells (MSCs) and explores their potential applications in the treatment and prognosis of OA, highlighting the value of lipidomics in understanding the pathogenic mechanisms of OA and in the development of novel therapeutic strategies.

Overall, these studies underscore the importance of lipidomic analysis in understanding the molecular mechanisms underlying OA and suggest that lipid composition may serve as a valuable target for future therapeutic interventions in OA management.

## Radiomics studies of OA

### Application of radiomics in early diagnosis of OA

Radiomic features have been increasingly utilized in the field of OA research to predict and diagnose the disease. Hirvasniemi et al. conducted a study focusing on distinguishing knees without and with OA using magnetic resonance imaging (MRI)-based radiomic features from the tibial bone [162]. They analyzed the right knees of 665 females from the Rotterdam Study and segmented the tibial bone using a combination of multi-atlas and appearance models. Similarly, Xie et al. introduced a new radiomics analysis for cartilage and subchondral bone to differentiate knees predisposed to posttraumatic OA after anterior cruciate ligament reconstruction from healthy knees [163]. In the analysis of MRI of subchondral bone, radiomic analysis is employed to identify KOA, which is crucial for understanding the pathological mechanisms of OA [164].

Yu et al. investigated the predictive value of infrapatellar fat pad (IPFP) radiomic features for incident radiographic knee OA (iROA) using data from the Osteoarthritis Initiative (OAI) [165]. They aimed to predict iROA diagnosis one year prior to its occurrence. In a related study, Ye et al. explored the radiomic signature of the IPFP for assessing KOA progression in older adults [166]. They found that radiomic alterations in the IPFP were associated with the severity and structural abnormalities of KOA in older adults. In addition to the IPFP, Villagran et al. focused on radiomic features of the medial meniscus to predict incident destabilizing meniscal tears in OA patients [167]. Li et al. explored the use of radiomics analysis of bone marrow edema in diagnosing KOA, while also developing an automatic grading model for KOA using plain radiograph radiomics, improved the efficiency and accuracy of the diagnosis [168]. Davey et al. conducted a systematic review and meta-analysis on the use of radiomics in predicting features of early OA of the knee, emphasizing the role of MRI findings in native knees [169]. Additionally, Jiang et al. explored MRI-based radiomics and delta-radiomics models of the patella to predict the radiographic progression of OA [170]. Overall, these studies highlight the potential of radiomics in improving the diagnosis, prediction, and assessment of OA progression, offering valuable insights into the current status and future perspectives of radiomics in OA research [171].

### Application of radiomics in prognosis assessment of OA

Additionally, Zhang et al. conducted a radiomics analysis of the patellofemoral joint to improve knee replacement risk prediction using data from the Multicenter Osteoarthritis Study [172]. They found that radiomics analysis of the patellofemoral joint could enhance the prediction of knee replacement risk. In a related study, Lin et al. developed a dynamic nomogram based on MRI-derived radiomics to predict knee pain improvement over two years for OA patients [173]. In a radiomics nomogram study based on MRI, researchers developed a predictive model to assess the impact of vitamin D treatment on patients with OA by analyzing radiomic features from MRI images [174]. Angelone et al. explored the potential of radiomics in predicting knee cartilage degeneration in patients with KOA [175]. The study utilized machine learning algorithms to analyze a multimodal dataset of MRI and CT scans, extracting radiomic features from cartilage segments and classifying knees as degenerated or healthy based on these features. Teoh et al. reviewed the imaging features of KOA for diagnosis and prognosis assessment [176]. The study emphasized the capability of machine learning methods in automating the diagnosis and prognosis of KOA through tasks such as knee joint localization, classification of OA severity, and prediction of disease progression.

### Integration of radiomics and deep learning

Recent advancements in medical imaging, particularly computed tomography, cone beam computed tomography, and MRI, have paved the way for the application of deep learning and radiomics in the diagnosis and management of OA. In the realm of KOA, machine learning approaches utilizing radiomic features have shown promise in distinguishing between knees with and without OA [162]. Li et al. developed an automatic grading model for knee OA using a plain radiograph radiomics model that combined anteroposterior and lateral images [177]. They utilized logistic regression to conduct the machine model for classification features. Le et al. introduced TMJOAI, an artificial web-based intelligence tool for the early diagnosis of temporomandibular joint osteoarthritis [178]. This tool utilizes machine learning algorithms to classify the health status of the temporomandibular joint in patients using clinical, biological, and jaw condyle radiomic markers.

Additionally, radiomic knee iROA prediction models have been developed using deep learning on MR images, showcasing the potential for automated and precise prediction of OA progression [165, 179]. The integration of deep learning approaches, such as convolutional neural networks, has further enhanced the efficacy of radiomic models in grading KOA based on plain radiographs [177]. Machine learning models based on MRI radiomics analysis have also been developed to diagnose KOA and predict the need for total knee replacement [180, 181]. Li et al. has developed a model that combines radiomics and neural networks, known as the Joint Space Radiomic Model, for predicting the onset of KOA [182]. This model integrates radiomic features of the femoral cartilage, tibial cartilage, and meniscus, and utilizes neural networks for modeling and prediction, demonstrating high accuracy in forecasting.

In conclusion, the combination of deep learning and radiomics in the field of OA holds great promise for improving diagnostic accuracy, treatment planning, and patient outcomes. The combination of radiomics and AI technology in OA research has shown tremendous potential, aiding in early diagnosis, precise assessment, and prognosis prediction of the disease. With continuous technological advancements and the improvement of databases, it is anticipated that this field will achieve more groundbreaking results in the coming years.

## Conclusion and prospects

In this review, we comprehensively showcase the latest advances and applications of omics research in OA through two detailed tables. Table 1 summarizes the contributions of various omics technologies in unveiling novel findings related to OA, while Table 2 summarizes how these findings can be concretely applied to the diagnosis, treatment, and prognosis of OA.

**Table 1:** A summary of new findings utilizing multi-omics approaches in osteoarthritis (OA) research

**Omics**	**Methods**	**Main comments**	**Ref**
Genomics	GWAS meta-analysis	52 of the identified SNVs represent previously unknown genetic risk factors for OA	[23]
	Multi-ancestry analyses in the Million Veteran Program and UK Biobank	Genetic variations associated with 27 OA loci-encompassing 10 novel loci-by integrating findings across diverse ancestries	[24]
	Genome-wide association of phenotypes based on clustering patterns of hand osteoarthritis	WNT9A, TGFα, RUNX2, COL27A1, ASTN2, IL11, and GDF5 had been identified as a potential novel pathogenic gene that may be involved in the pathogenic mechanisms of OA	[25]
	Whole genome chromosome conformation analysis on primary chondrocytes harvested from eight patients with knee osteoarthritis	SPRY4 and PAPPA are identified as candidate effector genes of OA. These genes may be involved in gene regulation through enhancer-promoter interactions	[26]
	High-throughput screening and bioinformatics tools analysis	Numerous candidate genes associated with OA have been identified and selected, such as ANO3, WWP2, Pitx1, Bhlhe40, Sh3bp4, and Unk	[27–29]
	Network pharmacology, molecular docking and in vitro experiments	Astragalus membranaceus can modulate the NRF2/HO-1 pathway through quercetin, thereby alleviating ferroptosis in chondrocytes and exerting a therapeutic effect on OA	[32]
	Microarray profiling and bioinformatic analysis	The upregulation of AMTN and DKK2, and the downregulation of MSLN in patients with OA is identified, highlighting their critical roles in OA-related pathological mineralization and bone remodeling	[40]
	In vitro experiments validation	Long non-coding RNA KLF3-AS1 represses autophagy and apoptosis of chondrocytes in OA	[49]
Transcriptomics	RNA sequencing technology and in vitro experiments validation	A specific small non-coding RNA, piRNA hsa_piR_019949 promotes chondrocyte anabolic metabolism by inhibiting the expression of the lncRNA NEAT1 in chondrocytes	[50]
	Validation of the OA model in cartilage-specific knockout mice (COL2^Cre^-miR-128a^loxp/loxp^; miR-128aKO) and in vitro experimental verification	MiRNA-128A has been shown to interrupt circadian rhythmicity signaling, accelerating the progress of OA	[52]
	Bioinformatics analysis	MiRNA-144 has been identified as having increased expression in both the early phase and end-stage of OA, highlighting its potential as a therapeutic target	[53]
	In vitro experiments validation	miRNA-140-3p has been associated with extensive downregulation of immune gene expression in an in vitro model of OA	[54]
	In vitro experiments and mouse model validation	Nanoparticles containing miRNA-224-5p have shown promise in balancing homeostasis, inhibiting cartilage degeneration, and alleviating synovial inflammation in OA	[57]
	Proteomic analysis in subchondral bone and bioinformatic analysis	A significant increase in SerpinA5 within the subchondral bone of OA patients has been observed, suggesting that the disruption of coagulation and complement pathways plays a role in the progression of OA and may provide a potential therapeutic target for the disease	[61]
	Urinary proteomics analysis	Urinary proteomics, through the identification of peptide biomarkers, has demonstrated the dysregulation of collagen synthesis and inflammation, and has also highlighted novel inflammatory markers for the identification and differentiation of distinct subtypes of inflammatory arthritis	[63]
	Quantitative proteomics analysis	Quantitative proteomics was used to reveal the protective effects of a compound called EDS against OA by attenuating inflammation and modulating immune responses	[64]
**Proteomics**	Proteomic analysis, nano-LC and Chip MS-MS analysis in serum	Elevated levels of vitronectin fragments (amino acids 381-397) have been identified in the serum of OA patients, which can interact with αVβ6 on human fibroblast-like synoviocytes to inhibit the activation of TGF-β1, potentially promoting fibrosis in OA	[66]
	Proteome data and pathway analysis thymus cell antigen 1 - fibroblast-like synoviocytes	An elevated expression of pyruvate dehydrogenase kinase 3 as a distinctive feature of proliferative THY1+ FLS in individuals with OA	[67]
	Proteomic analysis in synovial fluid	In patients with OA, there is an elevated presence of complement components such as C5, interleukin-6, and Cartilage Oligomeric Matrix Protein in synovial fluid	[69]
	Proteomic analysis in synovial fluid	The differential expression of 13 proteins was observed, suggesting a potential association with the progression of meniscal damage and OA	[70]
**Metabolomics**	SILAC-based proteomic and matrix-	Highlighted the impact of UDP-glucuronic acid and UDP-GlcNAc synthesis pathways on chondrogenic	[71]
	assisted laser desorption/ionization mass spectrometry imaging analysis in mesenchymal stromal cells	differentiation in OA cells, potentially affecting the production of cartilage ECM components	
	An ex-vivo equine cartilage explant OA model study	Identified differentially abundant metabolites, proteins, and ECM-derived neopeptides associated with OA pathogenesis	[72]
	Liquid chromatography-mass spectrometry and metabolomic analysis in articular cartilage and subchondral bone	Conducted studies comparing metabolomic profiles of healthy and OA human cartilage, revealing distinct metabolic differences between the two conditions	[74]
	A narrative review based on selected population-based metabolomics studies in OA	A systematic review of 32 population-based metabolomic studies utilizing plasma/serum, synovial fluid, cartilage, or subchondral bone samples has uncovered metabolic pathways implicated in OA, including energy metabolism, arginine and proline metabolism, taurine and hypotaurine metabolism, and glycerophospholipid metabolism	[78]
	Liquid chromatography/mass spectrometry-based metabolomics analysis in serum	Analysis of serum metabolomics in rats with OA revealed significant differences in metabolite levels, including decreased estradiol2 levels in the OA group	[80]
	Metabolomic analysis in serum and synovial fluid	Compared metabolites in synovial fluid and serum to identify potential biomarkers of joint injury in a mouse model of posttraumatic OA, shedding light on the pathophysiology of the disease	[85]
	^1^H NMR spectroscopy and quantified using CHENOMX metabolomics analysis software in synovial fluid	Focused on ^1^H NMR metabolic profiling of synovial fluid from patients with anterior cruciate ligament tears and hemarthrosis, highlighting the diagnostic potential of metabolomic analysis in OA	[86]
	Bioinformatics analysis	Utilized bioinformatics analysis to identify key biomarkers and immune infiltration in the synovial membrane of OA	[88]
	Proton-nuclear magnetic resonance (^1^H NMR) and metabolomics analysis in synovial fluid and synovial tissue	Conducted metabolomic profiling of synovial membrane to reveal biomarkers of synovial inflammation in OA patients	[89]
	Gas chromatography-time of flight/mass spectrometry-based metabonomics analysis in the urinary	The metabonomic pathogenesis of OA might be associated with disorders in amino acid metabolism, energy metabolism, fatty acid metabolism, vitamin B6 metabolism and nucleic acid metabolism	[90]
	Liquid chromatography-high resolution mass spectrometry, Human Metabolome Database, authentic standards and/or MS/MS database analysis in urinary	Urinary metabolomics has unveiled shared metabolic pathways and biomarkers between OA and other conditions, such as perturbation in glutamine metabolism, aiding in the enhanced comprehension of OA's pathological mechanisms and offering potential targets for the development of novel therapeutic strategies	[91]
	Illumina microarrays analysis in peripheral blood mononuclear cells DNA	DNA methylation patterns in peripheral blood mononuclear cells as potential predictors of radiographic progression in OA patients	[94]
	Quantified CpG methylation in human enhancers based on a public dataset and bioinformatics analysis	A genome-wide analysis revealed aberrant DNA methylation patterns in enhancers in knee and hip OA patients	[96]
**Epigenetics**	Integrative epigenetic analysis of OA cartilage using pyrosequencing, CRISPR/Cas9 editing, and targeted epigenome modulation	The genetic and epigenetic regulation of COLGALT2 has been investigated, demonstrating its association with OA genetic risk	[97]
	Integrated transcriptomic, epigenetic, and functional analysis of OA cartilage using public datasets and in vitro experiments	The methylation status of the CAMP gene promoter has been linked to chondrocyte apoptosis in OA patients	[99]
	In vitro experiments and mouse OA model validation	Inhibition of KDM7A/B histone demethylases shows protective effects against OA by restoring H3K79 methylation	[102]
	Genome-wide DNA methylation profiles of primary infrapatellar fat pad and blood samples, two-sample Mendelian randomization and colocalization analyses	Epigenomic profiling of the infrapatellar fat pad in OA patients has revealed genome-wide DNA methylation profiles	[103]
	In vitro experiments validation	The study reveals that matrix stiffness modulates H3K27me3 demethylation by the opening of mitochondrial permeability transition pores and the translocation of plant homeodomain finger protein 8, subsequently affecting the expression of Mmp13 and Bax genes, which exacerbates the progression of OA	[104]
	In vitro experiments and mouse OA model validation	Histone H3K9 methylation has been implicated in temporomandibular joint OA, with decreased levels observed in degenerated condylar articular cartilage in aged mice	[107]
	In vitro experiments and mouse OA model validation	By analyzing human and murine models, genetic variations in the DOT1L gene were found to correlate with OA and stature, and the histone-modifying enzyme DOT1L-mediated H3K79 methylation was linked to the Wnt signaling pathway	[108]
**Single-Cell Omics**	Single-cell RNA sequencing in mouse articular chondrocytes	Conducted a study on mouse articular chondrocytes and identified nine chondrocyte subtypes with distinct molecular profiles, highlighting injury-induced early molecular changes in response to joint trauma	[113]
	Single-cell RNA sequencing in human chondrocytes	Compared chondrocyte states in femoral cartilage between healthy individuals and OA patients, revealing differences in cell subtypes and proposing a novel classification of each subtype	[114]
	Single-cell and single-nuclei RNA sequencing of human IPFP and synovial tissues	Through single-cell RNA sequencing of the infrapatellar fat pad and synovium, and the creation of a comprehensive single-cell atlas, it was discovered that APOE exerts a deleterious impact on cartilage	[119]
	Single-cell RNA sequencing and single-cell assay for transposase-accessible chromatin sequencing data analysis	Single-cell analyses have been utilized to identify specific cell types contributing to the progression of KOA, such as anterior cruciate ligament fibroblasts	[121]
	Genome-wide association study and single-nucleus assay analysis	These analyses have also been instrumental in understanding the cellular origins of complex diseases like type 1 diabetes and identifying new therapeutic targets for OA	[122]
	RNA microarray and single-cell sequencing data analysis from gene expression omnibus database and scRNA-seq analysis in human knee synovial tissues and mouse OA model validation	It was found that six RNA modification-related genes (ADAMDEC1, IGHM, OGN, TNFRSF11B, SCARA3 and PTN) were identified as possible biomarkers of OA and RA pathogenesis	[124]
	Mass cytometry analysis in serum	Inflammatory arthritis, including OA, has been studied using deep single-cell proteomics analysis to identify insufficient PD-1 expression during active autoimmune responses	[125]
	16S ribosomal RNA amplicon sequencing of stool samples and multiplex cytokine analysis of blood samples	The presence of lipopolysaccharide in the blood was linked to obesity-related OA. This suggests a potential mechanism through which gut microbiota may influence the development of OA	[129]
	16S ribosomal RNA sequencing of stool samples	Examined the association between gut microbiota and elevated serum urate levels, suggesting that microbiota dysbiosis may modulate these levels, potentially impacting OA development	[131]
	16S ribosomal RNA amplicon sequencing on faecal samples and HPLC mass spectrometry analysis of plasma bile acids	Investigated the relationship between gut microbiota dysbiosis and hand synovitis prevalence, suggesting a potential role of bile acids as mediators in this association	[133]
**Microbiomics**	16S ribosomal RNA gene deep sequencing on cartilage samples from knee OA patients and hip OA patients	By analyzing cartilage samples from human knees and hips, it was found that compared to healthy controls, OA samples exhibited reduced microbial diversity, and there was an increase in functions related to lipopolysaccharide production, phosphatidylinositol signaling, and nitrogen metabolism, while the function of sphingolipid metabolism was decreased	[142]
	Lipidomic analyses of serum and joint synovial fluid from mouse OA model	Investigated the role of fatty acid composition and metabolic inflammation in post-traumatic OA, suggesting that systemic factors may contribute to OA pathogenesis	[148]
**Lipidomics**	Ultra-high performance liquid chromatography accurate mass high resolution mass spectrometry analysis in plasma samples from mouse OA model	Explored alterations in plasma lipidomic profiles in a mouse model of OA, aiming to identify biomarkers of pain and pathology	[150]
	Targeted mass spectrometry lipidomics and in vitro experiments validation	Focused on bioactive lipid families in an in vitro model of OA, demonstrating the involvement of specific lipids in the inflammatory response	[151]
	Untargeted lipidomic analysis of human sera and urine samples	Identified diagnostic lipidomic biomarkers for seropositive and seronegative rheumatoid arthritis, highlighting the potential of lipidomics in distinguishing different arthritic conditions	[155]
	Liquid chromatography/mass spectrometry-based label-free quantitative proteomic and lipidomic analysis of human intra-articular fat pad	Conducted a study that delved into the proteomic and lipidomic landscape of the infrapatellar fat pad in KOA, uncovering distinct molecular characteristics that could serve as potential diagnostic and therapeutic targets	[156]
	Matrix-assisted laser desorption/ionization mass spectrometry imaging and bottom-up proteomics of human cartilages	Investigated the differences in lipidomics and proteomics of cartilage between OA patients with and without type 2 diabetes, it was found that abnormal omega oxidation and fatty acid biosynthesis pathway may lead to imbalance of lipid metabolism	[157]
	Liquid chromatography-mass spectrometry analysis of human synovial fluid	Targeted lipidomics has revealed the activation of resolution pathways in human KOA, offering potential therapeutic targets for OA treatment	[160]
	Magnetic resonance imaging and a fast imaging employing steady-state acquisition sequence analysis of human knees	Conducted a study focusing on distinguishing knees without and with OA using magnetic resonance imaging (MRI)-based radiomic features from the tibial bone	[162]
**Radiomics**	Magnetic resonance imaging of human knee cartilage and subchondral bone	Introduced a new radiomics analysis for cartilage and subchondral bone to differentiate knees predisposed to posttraumatic OA after anterior cruciate ligament reconstruction from healthy knees	[163]
	Magnetic resonance imaging of human infrapatellar fat pad	Explored the radiomic signature of the IPFP for assessing KOA progression in older adults. They found that radiomic alterations in the IPFP were associated with the severity and structural abnormalities of KOA in older adults	[166]
	Magnetic resonance imaging of human meniscus	Focused on radiomic features of the medial meniscus to predict incident destabilizing meniscal tears in OA patients	[167]
	Magnetic resonance imaging of human bone marrow edema and cartilage injury	Explored the use of radiomics analysis of bone marrow edema in diagnosing KOA, while also developing an automatic grading model for KOA using plain radiograph radiomics, improved the efficiency and accuracy of the diagnosis	[168]
	Magnetic resonance imaging, computed Tomography and machine learning analysis of human knee	Explored the potential of radiomics in predicting knee cartilage degeneration in patients with KOA. The study utilized machine learning algorithms to analyze a multimodal dataset of MRI and CT scans, extracting radiomic features from cartilage segments and classifying knees as degenerated or healthy based on these features	[175]
	An artificial web-based intelligence tool, machine learning, radiomics analysis	Introduced TMJOAI, an artificial web-based intelligence tool for the early diagnosis of temporomandibular joint osteoarthritis. This tool utilizes machine learning algorithms to classify the health status of the temporomandibular joint in patients using clinical, biological, and jaw condyle radiomic markers	[178]
	Magnetic resonance imaging-based joint space radiomic model and neural networks integrative analysis of meniscus and femorotibial cartilage	Has developed a model that combines radiomics and neural networks, known as the Joint Space Radiomic Model, for predicting the onset of KOA. This model integrates radiomic features of the femoral cartilage, tibial cartilage, and meniscus, and utilizes neural networks for modeling and prediction, demonstrating high accuracy in forecasting	[182]

**Table 2:** A summary of clinical applications derived from osteoarthritis (OA) research findings

**Application domain**	**Methods **	**Main comments **	**Clinical significance **	**Ref **
**Diagnosis**	GWAS meta-analysis	The study identified a total of 100 independent and significantly associated risk single nucleotide variants (SNVs), with 60 of these SNVs correlating to more than one OA phenotype. Furthermore, 52 of the identified SNVs represent previously unknown genetic risk factors for the disease	These findings may serve as biomarkers for the early diagnosis of OA. They can be integrated with clinical symptoms and imaging studies to improve diagnostic accuracy.	[23]
	Multi-ancestry analyses in the Million Veteran Program and UK Biobank	Genetic variations associated with 27 OA loci-encompassing 10 novel loci-by integrating findings across diverse ancestries. Among these 27 loci, 476 single nucleotide polymorphisms were significantly correlated with OA. These observations corroborate previous findings and provide evidence that certain OA-associated regions are consistent across different human populations.	This helps improve the accuracy of diagnosis for patients of different ethnicities.	[24]
	Genome-wide association of phenotypes based on clustering patterns of hand OA	WNT9A has been identified as a potential novel pathogenic gene that may be involved in the pathogenic mechanisms of OA. In addition, several previously identified OA genetic loci appear to confer risk for the development of OA across multiple joints: these include loci for TGFα, RUNX2, COL27A1, ASTN2, IL11, and GDF5.	By creating phenotypes through clustering patterns of radiographic OA severity in different hand joints, this approach helps capture different OA subtypes and improves the accuracy of hand OA diagnosis.	[25]
	Whole genome chromosome conformation analysis on primary chondrocytes harvested from eight patients with KOA	Norbert Bittner and colleagues conducted a comprehensive genome-wide chromatin conformation analysis (Hi-C) on primary chondrocytes harvested from eight patients with KOA, generating a detailed map that correlates chromosomal architecture with genomic sequence. This study led to the identification of two novel candidate effector genes, SPRY4 and PAPPA (pregnancy-associated plasma protein A), these genes may be involved in gene regulation through enhancer-promoter interactions.	This funding identifies novel candidate effector genes for OA, thereby enhancing our understanding of the disease.	[26]
	In vitro experiments validation	Conducted a study using cytokine-treated tissue-engineered cartilage as an in vitro model of OA to perform transcriptomic analysis.	This approach allowed for the identification of key gene expression changes associated with OA development, specifically in the catabolic aspect of inflammation induce OA.	[33]
	Transcriptome analysis of ontogenetic and regenerating scale of zebrafish	Investigated the transcriptome of regenerating zebrafish scales to identify genes relevant to human bone diseases, including OA.	The study identified differentially expressed genes enriched for pathways related to ECM, ossification, and cell adhesion, providing potential targets for further research.	[34]
	RNA sequencing technology and mouse OA model validation	Focused on transcriptomic analyses in human and murine infrapatellar fat pads to identify common profibrotic changes in OA. This study shed light on the molecular alterations occurring in adipose tissue in the context of OA.	This discovery helps to identify commonly dysregulated pathways during the early phases of the disease	[35]
	Whole-transcriptome sequencing and ceRNA interaction network of temporomandibular joint osteoarthritis (TMJOA)	Explored the transcriptomic landscape of temporomandibular joint OA, highlighting the potential of noncoding RNAs as clinical biomarkers and providing insights into their functional roles in OA.	This study showed the potential of lncRNAs, circRNAs, miRNAs, and mRNAs as clinical biomarkers and provided transcriptomic insights into their functional roles in TMJOA, which contribute to improving the accuracy of diagnosis.	[36, 37]
	Deep RNA sequencing analysis of OA knee cartilage	Conducted a large-scale study on lncRNA expression, isoform switching, and alternative splicing in OA, revealing novel genomic complexities associated with the disease.	This study identified differential splicing in 209 genes associated with OA, involving pathways such as extracellular matrix, proteoglycans, and integrin interactions. It highlights the impact of splicing and gene expression changes on the disease, providing new insights into the mechanisms and diagnosis of OA.	[45]
	RNA sequencing analysis of OA plasma	A distinct signature of circulating miRNAs has been identified in early radiographic knee OA, suggesting their potential as biomarkers for early disease detection.	The specific expression patterns of these miRNAs provide valuable insights into the early diagnosis of KOA.	[55]
	Mass spectrometry-based techniques analysis of OA patients’ meniscal tissue	Focused on proteomic analysis of meniscus cartilage in OA patients. By characterizing the proteomic profiles of meniscal tissue and developing an assay for ECM proteins, this study contributes to understanding the molecular changes in OA-affected cartilage.	This study analyzed the proteomic profiles of meniscal cartilage in OA patients, uncovering molecular changes associated with OA. It provides valuable insights for early diagnosis.	[60]
	Proximity extension array analysis of OA patients’ serum	Conducted a study to assess serum levels of inflammatory markers in patients with KOA using Proximity Extension Array technology. Their findings aimed to evaluate the associations between these markers and clinical pain intensity in KOA patients.	This provides novel molecular insights into the pain mechanisms of KOA and facilitates the identification of potential inflammation-related diagnostic biomarkers.	[62]
	Urinary proteomics analysis	Urinary proteomics, through the identification of peptide biomarkers, has demonstrated the dysregulation of collagen synthesis and inflammation, and has also highlighted novel inflammatory markers for the identification and differentiation of distinct subtypes of inflammatory arthritis.	This provides a novel approach for the early diagnosis and classification of inflammatory arthritis.	[63]
	Proteomic analysis of OA patients’ synovial tissue	Researchers have identified proteins that are differentially expressed in synovial membrane, linking menopause with the progression of OA and potentially leading to novel diagnostic and therapeutic strategies.	It uncovers the potential molecular mechanisms linking menopause and OA, providing new scientific evidence for early diagnosis and personalized treatment of the disease.	[65]
	Proteomic analysis in synovial fluid	Proteomic biomarkers of meniscal disease have been identified from synovial fluid samples derived from control knees as well as knees exhibiting varying severities of meniscal injury and OA. The differential expression of 13 proteins was observed, suggesting a potential association with the progression of meniscal damage and OA.	These findings may pave the way for the exploration of synovial fluid biomarkers in meniscal pathology.	[70]
	An ex-vivo equine cartilage explant OA model study	Conducted an ex-vivo equine cartilage explant OA model study, identifying differentially abundant metabolites, proteins, and ECM-derived neopeptides associated with OA pathogenesis.	This study offers valuable insights into the pathogenesis of early OA and highlights novel clinical biomarkers for advancing the understanding of disease progression.	[72]
	Liquid chromatography/mass spectrometry-based metabolomics analysis in serum	Serum metabolomics has shown promise in distinguishing individuals with OA from healthy controls. Analysis of serum metabolomics in rats with OA revealed significant differences in metabolite levels, including decreased estradiol2 levels in the OA group.	These findings not only enhance the understanding of the metabolic characteristics of OA but also suggest that certain metabolites (e.g., estradiol) may serve as potential biomarkers, providing new avenues for early screening of OA.	[80]
	Metabolomic analysis in serum and synovial fluid	Compared metabolites in synovial fluid and serum to identify potential biomarkers of joint injury in a mouse model of posttraumatic OA, shedding light on the pathophysiology of the disease.	This study offers new avenues for the early diagnosis and disease monitoring of post-traumatic osteoarthritis.	[85]
	^1^H NMR spectroscopy and quantified using CHENOMX metabolomics analysis software in synovial fluid	Focused on ^1^H NMR metabolic profiling of synovial fluid from patients with anterior cruciate ligament tears and hemarthrosis, highlighting the diagnostic potential of metabolomic analysis in OA.	This highlights the potential of metabolomics in the early diagnosis and disease monitoring of OA.	[86]
	H-NMR analysis of synovial fluid collected from OA horses	Demonstrated that the synovial fluid metabolome can differentiate between healthy joints and OA-affected joints in horses, showcasing the utility of metabolomics in disease diagnosis.	This study provides novel biomarkers for the early diagnosis of OA.	[87]
	Proton-nuclear magnetic resonance (^1^H NMR) and metabolomics analysis in synovial fluid and synovial tissue	Conducted metabolomic profiling of synovial membrane to reveal biomarkers of synovial inflammation in OA patients.	Their study shed light on the metabolic alterations associated with OA pathogenesis, providing valuable information for the development of targeted therapeutic strategies.	[89]
	Gas chromatography-time of flight/mass spectrometry-based metabonomics analysis in the urinary	Detected the urine of rats with OA via gas chromatography - time-of-flight/mass spectrometry (GC-TOF/MS), and discovered that the metabonomic pathogenesis of OA might be associated with disorders in amino acid metabolism, energy metabolism, fatty acid metabolism, vitamin B6 metabolism and nucleic acid metabolism.	The abnormalities in these metabolic pathways may serve as potential diagnostic biomarkers or therapeutic targets, providing a scientific basis for early detection and accurate diagnosis of the disease.	[90]
	Illumina microarrays analysis in peripheral blood mononuclear cells DNA	Recent studies have delved into the epigenetic mechanisms underlying OA pathogenesis, particularly focusing on DNA methylation patterns in peripheral blood mononuclear cells as potential predictors of radiographic progression in OA patients.	This study suggests that models based on PBMC DNA methylation hold promise as potential biomarkers for predicting OA progression. This finding provides valuable clinical insights for early assessment and personalized treatment of OA.	[94]
	Integrative epigenetic analysis of OA cartilage using pyrosequencing, CRISPR/Cas9 editing, and targeted epigenome modulation	The genetic and epigenetic regulation of COLGALT2 has been investigated, demonstrating its association with OA genetic risk.	COLGALT2 has the potential to serve as a novel biomarker for predicting osteoarthritis risk and managing the disease, thereby expanding our understanding of the pathogenesis of OA.	[97]
	Epigenome-wide association study of cartilage collected from OA patients	Epigenome-wide studies have provided a comprehensive view of OA in primary tissues, offering insights into the epigenetic landscape of the disease.	The findings provide enhanced insights into epigenetic mechanisms underlying OA in primary tissues	[100]
	Genome-wide buffy coat DNA methylation patterns from the Osteoarthritis Biomarkers Consortium	Machine learning models based on peripheral blood DNA methylation have been developed to predict knee OA progression, addressing the need for accurate biomarkers in OA clinical research.	These studies have unveiled the epigenetic characteristics of OA, offering novel insights into its etiology, pathogenesis, and progression.	[101]
	Genome-wide DNA methylation profiles of primary infrapatellar fat pad and blood samples, two-sample Mendelian randomization and colocalization analyses	Epigenomic profiling of the infrapatellar fat pad in OA patients has revealed genome-wide DNA methylation profiles, providing valuable insights into the epigenetic regulation of OA.	The study revealed genome-wide DNA methylation patterns in the infrapatellar fat pad of OA patients, providing important insights into the epigenetic regulatory mechanisms of OA and aiding in the identification of potential diagnostic and therapeutic targets.	[103]
	Bottom-up LC-MS/MS analyses of chondrocytes collected from OA patients	The dysregulation of histone modifications, including histone methylation and acetylation, can lead to aberrant gene expression patterns in OA, contributing to disease development and progression.	This study provides a new perspective on the molecular pathogenesis of OA, contributing to the improvement of diagnostic and therapeutic strategies for OA patients.	[106]
	In vitro experiments and mouse OA model validation	Histone H3K9 methylation has been implicated in temporomandibular joint OA, with decreased levels observed in degenerated condylar articular cartilage in aged mice.	Changes in H3K9 methylation levels may serve as a potential molecular biomarker for TMJOA, offering a promising indicator for early diagnosis.	[107]
	Histone analysis from human cartilage	The study found that OA chondrocytes increase IL-1β and IL-8 production, which are epigenetically regulated, and that these cytokines stimulate matrix metalloproteinases and aggrecanase synthesis, leading to ECM degradation. Thus, examining histone post-translational modifications is essential for understanding the epigenetic control of inflammatory factors in OA.	The study highlights the critical role of histone post-translational modifications in the pathogenesis and progression of OA, offering new insights into its diagnosis and treatment.	[109]
	Single-cell RNA sequencing in mouse articular chondrocytes	Conducted a study on mouse articular chondrocytes and identified nine chondrocyte subtypes with distinct molecular profiles, highlighting injury-induced early molecular changes in response to joint trauma.	It enhanced understanding of early molecular changes and chondrocyte diversity in joint trauma. It could aid in early diagnosis.	[113]
	Single-cell RNA sequencing in human chondrocytes	Compared chondrocyte states in femoral cartilage between healthy individuals and OA patients, revealing differences in cell subtypes and proposing a novel classification of each subtype.	This study helps identify specific chondrocyte changes associated with early-stage OA, thereby improving the accuracy of OA diagnosis.	[114]
	Single-cell RNA sequencing of cartilage collected from OA patients	Integrated multi-omics data to unveil inflammatory and prehypertrophic cell populations as key contributors to knee cartilage degeneration in OA, defining 11 chondrocyte populations, including pre-inflammatory and inflammatory chondrocyte populations.	The study can help in understanding the immune microenvironment in PTOA. This information could be used to develop biomarkers for earlier and more accurate detection of PTOA.	[116]
	Single-cell RNA sequencing analysis of fibroblasts	Single-cell analyses have been utilized to identify specific cell types contributing to the progression of KOA, such as anterior cruciate ligament fibroblasts.	The clinical significance of this study lies in its identification of specific cell types, such as anterior cruciate ligament fibroblasts, involved in the progression of KOA, thereby deepening the understanding of the underlying disease mechanisms.	[121]
	RNA microarray and single-cell sequencing data analysis from gene expression omnibus database and scRNA-seq analysis in human knee synovial tissues and mouse OA model validation	It was found that six RNA modification-related genes (ADAMDEC1, IGHM, OGN, TNFRSF11B, SCARA3 and PTN) were identified as possible biomarkers of OA and RA pathogenesis.	This research revealed the significance of RNA modification-related genes in the development of OA and RA pathogenesis, thereby providing a novel research direction for understanding the mechanisms, diagnosis, and treatment of OA.	[124]
	A deep single-cell proteomics	A study used deep single-cell proteomics analysis to identify insufficient PD-1 expression during active autoimmune responses.	This finding may offer new perspectives for the diagnosis of autoimmune diseases.	[125]
	16S ribosomal RNA sequencing analysis of stool samples from OA patients	Conducted a study on the Xiangya Osteoarthritis Study participants to examine the association between gut microbiome and symptomatic hand OA. Their findings suggest a potential link between gut microbiota and the presence of symptomatic hand OA.	This study may help understand the role of microbiome in the development of this common condition.	[127]
	Large-scale genome-wide association studies and two-sample Mendelian randomization analysis	Explored the causal role of gut microbiota in the development of OA, further supporting the idea that gut microbiota may play a role in the pathogenesis of OA.	This study found that several microbial taxa were causally associated with diverse joint OA. The results enhanced our understanding of gut microbiota in the pathology of OA.	[128]
	16S ribosomal RNA amplicon sequencing on faecal samples and HPLC mass spectrometry analysis of plasma bile acids	Investigated the relationship between gut microbiota dysbiosis and hand synovitis prevalence, suggesting a potential role of bile acids as mediators in this association.	This study suggests that bile acids may serve as potential diagnostic biomarkers for related diseases, offering new perspectives for further exploration of the pathogenesis of inflammatory diseases.	[133]
	Lipidomic analyses of serum and joint synovial fluid from mouse OA model	Investigated the role of fatty acid composition and metabolic inflammation in post-traumatic OA, suggesting that systemic factors may contribute to OA pathogenesis.	This study reveals that fatty acid composition and systemic metabolic inflammation may be key risk factors for obesity-associated OA.	[148]
	Matrix-assisted laser desorption ionization - mass spectrometry imaging of human synovial membranes	Identified a distinct lipidomic profile in the OA synovial membrane using mass spectrometry imaging, highlighting the importance of lipid composition in OA pathology.	This study, utilizing mass spectrometry imaging, identified distinct lipidomic characteristics in the synovial membrane of OA, highlighting the critical role of lipid composition in OA pathogenesis and suggesting potential novel biomarkers for its diagnosis.	[149]
	Ultra-high performance liquid chromatography accurate mass high resolution mass spectrometry analysis in plasma samples from mouse OA model	Explored alterations in plasma lipidomic profiles in a mouse model of OA, aiming to identify biomarkers of pain and pathology.	This study investigated alterations in plasma lipidomic profiles in a mouse model of OA with the aim of identifying potential biomarkers associated with pain and pathology. It provides novel insights into early diagnosis and therapeutic strategies for OA.	[150]
	Untargeted lipidomic analysis of human sera and urine samples	Identified diagnostic lipidomic biomarkers for seropositive and seronegative rheumatoid arthritis, highlighting the potential of lipidomics in distinguishing different arthritic conditions.	It identified a panel of ten serum lipids as potential biomarkers that can differentiate RA from OA and SLE, regardless of seropositivity.	[155]
	Liquid chromatography/mass spectrometry-based label-free quantitative proteomic and lipidomic analysis of human intra-articular fat pad	Conducted a study that delved into the proteomic and lipidomic landscape of the infrapatellar fat pad in KOA.	It uncovered that the proteomic and lipidomic of the infrapatellar fat pad in KOA distinct molecular characteristics that could serve as potential diagnostic and therapeutic targets	[156]
	Lipidomic and adipokine analyses of serum and synovial fluid collected from OA mouse	By examining the lipidomic profiles in plasma and synovial fluid, researchers have been able to predict obesity-related OA, synovitis, and wound healing, suggesting that lipidomic signatures may play a role in the early diagnosis and prognosis of OA.	This study extends our understanding of the links of FAs with OA, synovitis and wound healing, and reports newly identified serum and synovial fluid FAs as predictive biomarkers of OA in obesity.	[158]
	Magnetic resonance imaging and a fast imaging employing steady-state acquisition sequence analysis of human knees	Conducted a study focusing on distinguishing knees without and with OA using MRI-based radiomic features from the tibial bone. They analyzed the right knees of 665 females from the Rotterdam Study and segmented the tibial bone using a combination of multi-atlas and appearance models.	This study utilized MRI to extract radiomic features of the tibial bone, successfully distinguishing normal knees from those with OA. It provides novel imaging-based evidence for the early diagnosis and precise classification of OA, offering significant clinical value.	[162]
	Magnetic resonance imaging-based joint space radiomic model	Introduced a new radiomics analysis for cartilage and subchondral bone to differentiate knees predisposed to posttraumatic OA after anterior cruciate ligament reconstruction from healthy knees.	The radiomics features of the cartilage and subchondral bone may be able to provide powerful tools with more sensitive detection.	[163]
	Machine learning-based support vector machine models analysis	In the analysis of MRI of subchondral bone, radiomic analysis is employed to identify KOA, which is crucial for understanding the pathological mechanisms of OA.	This fundings suggested that our MRI-based radiomics models can be used as biomarkers for the classification of OA and are superior to the conventional structural parameter-based model.	[164]
	U-Net was utilized for automatic segmentation of the infrapatellar fat pad (IPFP) in combination with radiomic and clinical features.	Investigated the predictive value of infrapatellar fat pad (IPFP) radiomic features for incident radiographic knee OA (iROA) using data from the Osteoarthritis Initiative (OAI). They aimed to predict iROA diagnosis one year prior to its occurrence.	This funding demonstrated that radiomic features of the IPFP are predictive of iROA 1 year prior to the diagnosis, suggesting that IPFP radiomic features can serve as an early quantitative prediction biomarker of iROA.	[165]
	Magnetic resonance imaging of human infrapatellar fat pad	Explored the radiomic signature of the IPFP for assessing KOA progression in older adults. They found that radiomic alterations in the IPFP were associated with the severity and structural abnormalities of KOA in older adults.	The radiomic signature may be a reliable biomarker to detect IFP abnormality of KOA.	[166]
	Magnetic resonance imaging of human meniscus	Villagran et al. focused on radiomic features of the medial meniscus to predict incident destabilizing meniscal tears in OA patients.	The use of radiomic features provides sensitive and quantitative measures of meniscal alterations, allowing us to intervene and prevent destabilizing meniscal tears.	[167]
	Magnetic resonance imaging of human bone marrow edema and cartilage injury	Explored the use of radiomics analysis of bone marrow edema in diagnosing KOA, while also developing an automatic grading model for KOA using plain radiograph radiomics, improved the efficiency and accuracy of the diagnosis.	The MRI-bone marrow edema-based radiomics-clinical nomogram model showed good performance in diagnosing early OA.	[168]
	Using Osteoarthritis Initiative data, features were selected through LASSO and models were constructed using SVM.	Jiang et al. explored MRI-based radiomics and delta-radiomics models of the patella to predict the radiographic progression of OA.	The MRI-based radiomics models of the patella all showed good predictive performance in predicting the radiographic progression of OA.	[170]
	Using a multimodal dataset of MRI and CT scans from KOA patients	Explored the potential of radiomics in predicting knee cartilage degeneration in patients with KOA. The study utilized machine learning algorithms to analyze a multimodal dataset of MRI and CT scans, extracting radiomic features from cartilage segments and classifying knees as degenerated or healthy based on these features.	Results demonstrate high accuracy in knee OA classification using radiomics, showcasing its potential for early disease detection and personalized treatment approaches.	[175]
	Radiomic analysis of knee joint radiographs, combined with logistic regression modeling	Developed an automatic grading model for knee OA using a plain radiograph radiomics model that combined anteroposterior and lateral images. They utilized logistic regression to conduct the machine model for classification features.	A radiomic model can improve the grading of knee OA and assist in diagnosis and treatment.	[177]
	Machine learning based TMJOAI analysis of TMJOA patients	Introduced TMJOAI, an artificial web-based intelligence tool for the early diagnosis of temporomandibular joint osteoarthritis. This tool utilizes machine learning algorithms to classify the health status of the temporomandibular joint in patients using clinical, biological, and jaw condyle radiomic markers.	This tool enables the early and accurate diagnosis of TMJOA.	[178]
	Machine learning models based on MRI radiomics analysis	Machine learning models based on MRI radiomics analysis have also been developed to diagnose KOA and predict the need for total knee replacement.	This study enhances the accuracy of early diagnosis of KOA and predicts the need for total knee replacement through MRI radiomics and machine learning models.	[180, 181]
	Magnetic resonance imaging-based joint space radiomic model and neural networks integrative analysis of meniscus and femorotibial cartilage	Developed a model that combines radiomics and neural networks, known as the Joint Space Radiomic Model, for predicting the onset of KOA. This model integrates radiomic features of the femoral cartilage, tibial cartilage, and meniscus, and utilizes neural networks for modeling and prediction, demonstrating high accuracy in forecasting.	This provides significant clinical value for the early diagnosis and personalized intervention of KOA.	[182]
	Network pharmacology, molecular docking and in vitro experiments	Astragalus membranaceus can modulate the NRF2/HO-1 pathway through quercetin, thereby alleviating ferroptosis in chondrocytes and exerting a therapeutic effect on OA.	GRS can facilitate the discovery of new biological pathways involved in OA and the identification of novel therapeutic targets.	[32]
**Therapy **	Genome-wide transcriptomic analysis of OA synovial macrophages	Wang et al. investigated the transcriptomic profile of OA synovial macrophages, revealing a tolerized phenotype compounded by a weak corticosteroid response.	This finding may help explain the limited efficacy of corticosteroid treatments in improving long-term OA prognosis.	[39]
	Microarray profiling and bioinformatic analysis	Utilized microarray profiling and bioinformatic analyses to discern the differential expression of genes in OA patients. They identified the upregulation of AMTN and DKK2, and the downregulation of MSLN, highlighting their critical roles in OA-related pathological mineralization and bone remodeling.	These findings suggest that targeting mineralization and inflammatory pathways could offer novel therapeutic avenues for OA management.	[40]
	Network biology approach analysis	Propose data integration as a useful strategy for creating lncRNA networks in arthritis, focusing on chondrocytes in OA and fibroblast-like synoviocytes in rheumatoid arthritis.	By employing integrative computational biology, disease-specific regulatory networks were constructed, offering valuable insights into disease mechanisms, potential therapeutic targets, and novel treatment strategies.	[46]
	In vitro experiments validation	Investigate the molecular mechanism of the lncRNA KLF3-AS1, derived from mesenchymal stem cell exosomes, in repressing autophagy and apoptosis of chondrocytes in OA.	This study suggests that MSC-Exo-mediated KLF3-AS1 may be a potential therapeutic target for OA.	[49]
	RNA sequencing technology and in vitro experiments validation	Focus on a specific small non-coding RNA, piRNA hsa_piR_019949, which promotes chondrocyte anabolic metabolism by inhibiting the expression of the lncRNA NEAT1 in chondrocytes.	PiRNA hsa_piR_019949 has demonstrated significant potential in promoting chondrocyte proliferation and enhancing extracellular matrix synthesis in patients with OA, primarily through the inhibition of the long non-coding RNA NEAT1. Synthetic mimics of hsa_piR_019949 represent a promising therapeutic strategy, offering a novel approach for the intervention and treatment of OA.	[50]
	Validation of the OA model in cartilage-specific knockout mice (COL2^Cre^-miR-128a^loxp/loxp^; miR-128aKO) and in vitro experimental verification	MiRNA-128A has been shown to interrupt circadian rhythmicity signaling, accelerating the progress of OA.	This emerging pathway may highlight the therapeutic potential of miR-128a KO for warding off OA.	[52]
	Bioinformatics analysis	MiRNA-144 has been identified as having increased expression in both the early phase and end-stage of OA, highlighting its potential as a therapeutic target.	miRNA-144 may serve as a potential therapeutic target, and modulating its expression holds promise for intervening in disease progression, offering a novel strategy for the treatment of OA.	[53]
	In vitro experiments validation	MiRNA-140-3p has been associated with extensive downregulation of immune gene expression in an in vitro model of OA, further emphasizing the role of miRNAs in OA pathogenesis.	MiRNA-140-3p may serve as a crucial molecular biomarker for investigating the pathogenesis of OA, facilitating the development of novel therapeutic approaches.	[54]
	In vitro experiments and mouse OA model validation	Nanoparticles containing miRNA-224-5p have shown promise in balancing homeostasis, inhibiting cartilage degeneration, and alleviating synovial inflammation in OA, highlighting the therapeutic potential of miRNA-based interventions.	Nanoparticles loaded with miRNA-224-5p effectively regulate joint homeostasis, inhibit cartilage degeneration, and alleviate synovial inflammation, thereby contributing to the multifaceted improvement of OA pathology.	[57]
	Proteomic analysis in subchondral bone and bioinformatic analysis	A significant increase in SerpinA5 within the subchondral bone of OA patients has been observed, suggesting that the disruption of coagulation and complement pathways plays a role in the progression of OA and may provide a potential therapeutic target for the disease.	It provides a promising therapeutic target of OA.	[61]
	Quantitative proteomics analysis	Quantitative proteomics was used to reveal the protective effects of a compound called EDS against OA by attenuating inflammation and modulating immune responses.	It offers a new potential therapeutic option for the treatment of OA.	[64]
	Olink proteomic panel analysis of OA patients’ fibroblasts	Highlight the significance of obesity in both load-bearing and non-load bearing joints in changing the inflammatory molecular endotype OA synovial fibroblasts, providing a rational for the therapeutic targeting of specific synovial fibroblasts subsets in specific patient populations with arthritic conditions.	This contributes to the development of more precise therapeutic strategies based on the individual pathological characteristics of patients (such as obesity-related inflammatory mechanisms), thereby improving the symptoms of patients with different types of arthritis more effectively.	[68]
	In vitro experiments and mouse OA model validation	Histone demethylases have also been implicated in OA pathogenesis, with inhibition of KDM7A/B histone demethylases showing protective effects against OA by restoring H3K79 methylation.	The study reveals the role of KDM7A/B histone demethylases in the pathogenesis of OA and highlights the protective effects of their inhibition. This research opens new avenues for OA treatment.	[102]
	In vitro experiments and mouse OA model validation	By analyzing human and murine models, genetic variations in the DOT1L gene were found to correlate with OA and stature, and the histone-modifying enzyme DOT1L-mediated H3K79 methylation was linked to the Wnt signaling pathway.	It offered a novel therapeutic target for epigenetic-based treatments of OA.	[108]
	Genome-wide association study and single-nucleus assay for transposase-accessible chromatin with sequencing of OA patients	These analyses have also been instrumental in understanding the cellular origins of complex diseases like type 1 diabetes and identifying new therapeutic targets for OA.	This analysis is of great significance in understanding the cellular origins of complex diseases, such as type 1 diabetes, and contributes to the identification of novel therapeutic targets for OA.	[122]
	Mouse OA model validation	The study found that the gut microbiome may directly affect cartilage health through the circulatory system. Dietary adjustments, probiotics and prebiotics use, and fecal microbiota transplantation might exert therapeutic effects on OA by inhibiting cartilage catabolism or apoptosis.	This study suggests that interventions such as dietary modifications, the use of probiotics and prebiotics, as well as fecal microbiota transplantation, may have therapeutic potential for OA by inhibiting cartilage degradation or apoptosis.	[144–147]
	Targeted mass spectrometry lipidomics and in vitro experiments validation	Focused on bioactive lipid families in an in vitro model of OA, demonstrating the involvement of specific lipids in the inflammatory response.	These findings provide potential targets for understanding the inflammatory mechanisms of OA and developing anti-inflammatory therapeutic strategies.	[151]
	Lipidyzer™ platform and LC-MS/MS platform analysis of blood samples collected from OA patients	The use of lipidomics to predict the response to prednisolone treatment in patients with inflammatory hand osteoarthritis indicates that lipidomic features may aid in the implementation of personalized medicine.	This suggest that the patients' lipid profile may improve the discriminative accuracy of the prediction of prednisolone treatment response in patients with inflammatory hand OA compared to prediction by commonly measured patient characteristics alone.	[159]
	Liquid-chromatography mass spectrometry analysis of lipid mediators collected from OA patients	Targeted lipidomics has revealed the activation of resolution pathways in human KOA, offering potential therapeutic targets for OA treatment.	A better understanding of these pathways could guide us to more effective therapeutic approaches to inhibit inflammation and further structural damage in OA.	[160]
	High-throughput screening and bioinformatics tools analysis	Numerous candidate genes associated with OA have been identified and selected, such as ANO3, WWP2, Pitx1, Bhlhe40, Sh3bp4, and Unk.	These findings not only reveal how specific genetic variations increase the risk of OA by affecting the biological functions of chondrocytes and the integrity of joint tissue but also point to new directions for research to further understand how these variations influence the development and progression of the disease.	[27–29]
	RNA sequencing analyses of rat OA model	Investigated dysregulated mechanotransduction and ECM pathways in a rat model of OA through transcriptomic analyses of joint tissues.	This study identified key pathways involved in OA development, further elucidating the molecular mechanisms underlying this disease.	[38]
**Prognosis **	Circadian time series proteomics of mouse OA model cartilage	Explored the daily dynamics in cartilage physiology using circadian time series proteomics. By analyzing the proteomic changes in cartilage over time, this study provides insights into the temporal regulation of proteins involved in OA progression.	This finding provided a foundation for optimizing treatment strategies and improving disease prognosis based on circadian rhythms.	[58]
	iTRAQ-integrated liquid chromatography-tandem mass spectrometry of guinea pigs’ tibial subchondral bone	Conducted a differential proteomic analysis of tibial subchondral bone in guinea pigs with spontaneous OA to investigate molecular alterations in early OA. Their findings identified significant differences in protein expression between male and female guinea pigs, highlighting potential sex-specific mechanisms in OA development.	The study revealed significant differences in protein expression between male and female guinea pigs, highlighting potential sex-specific mechanisms underlying the pathogenesis of OA and offering new insights for developing personalized therapeutic strategies tailored to sex differences.	[59]
	Proteomic analysis, nano-LC and Chip MS-MS analysis in serum	Elevated levels of vitronectin fragments (amino acids 381-397) have been identified in the serum of OA patients, which can interact with αVβ6 on human fibroblast-like synoviocytes to inhibit the activation of TGF-β1, potentially promoting fibrosis in OA.	This finding highlights the potential role of vitronectin fragments in the pathogenesis of OA, offering new insights into the mechanisms underlying OA-related fibrosis.	[66]
	Proteome data and pathway analysis in thymus cell antigen 1 - fibroblast-like synoviocytes	A study has meticulously analyzed the metabolic, proteomic, and functional characteristics of THY1+ fibroblast-like synoviocytes in OA patients, uncovering an elevated expression of pyruvate dehydrogenase kinase 3 as a distinctive feature of proliferative THY1+ FLS in these individuals and reveals how metabolic changes in synovial fibroblasts contribute to OA.	This discovery provides new insights into the pathological mechanisms of OA, highlighting the critical role of metabolic reprogramming in synovial fibroblasts during disease progression.	[67]
	SILAC-based proteomic and matrix-assisted laser desorption/ionization mass spectrometry imaging analysis in mesenchymal stromal cells	Highlighted the impact of UDP-glucuronic acid and UDP-GlcNAc synthesis pathways on chondrogenic differentiation in OA cells, potentially affecting the production of cartilage ECM components. This emphasizes the importance of metabolic pathways in maintaining cartilage integrity.	This reveals the critical role of metabolic pathways in maintaining cartilage integrity and provides a new perspective on the pathological mechanisms of OA.	[71]
	Integrative analysis of transcriptomics and global metabolomics of murine epiphyseal hip cartilage before and after injury	Investigated the metabolic signature of articular cartilage post-injury using an integrative analysis of transcriptomics and metabolomics, shedding light on the metabolic changes in cartilage tissue following mechanical injury.	This study elucidates the significant metabolic changes in articular cartilage following mechanical injury, offering a new perspective on the critical role of early metabolic states in driving tissue damage and contributing to the progression of OA.	[73]
	Liquid chromatography-mass spectrometry and metabolomic analysis in articular cartilage and subchondral bone	Conducted studies comparing metabolomic profiles of healthy and OA human cartilage, revealing distinct metabolic differences between the two conditions. This comparative analysis highlighted specific metabolic pathways with unique regulation patterns in OA cartilage.	This study identified the unique regulatory patterns of specific metabolic pathways in OA cartilage, providing valuable insights into the underlying pathological mechanisms of OA.	[74]
	Ultraperformance liquid chromatography tandem quadrupole time-of-flight mass spectrometry analysis of OA patients’ cartilage	Metabolomic methodologies have the capacity to elucidate the underlying mechanisms of osteophyte formation in OA, revealing metabolic alterations associated with this pathological development, including changes in amino acids, sulfonic acids, glycerophospholipids, and fatty acyls. These metabolites are implicated in physiological and pathological processes such as cartilage dissolution, disruption of boundary layers, and the triggering of self-repair mechanisms. Notably, phenylalanine metabolism is significantly correlated with this degenerative process.	This findings provided a direction for targeted metabolomic study and an insight into further reveal the molecular mechanisms of ostophyte formation.	[75]
	A narrative review based on selected population-based metabolomics studies in OA	A systematic review of 32 population-based metabolomic studies utilizing plasma/serum, synovial fluid, cartilage, or subchondral bone samples has uncovered metabolic pathways implicated in OA, including energy metabolism, arginine and proline metabolism, taurine and hypotaurine metabolism, and glycerophospholipid metabolism.	These findings contribute to a better understanding of the pathogenesis of OA, facilitate the classification of OA patients into distinct endotypes, and support the development of precision medicine tools for OA management.	[78]
	Nuclear magnetic resonance-based metabolomic analysis of rat OA model serum samples	Anterior cruciate ligament damage could significantly disrupt energy metabolism.	The findings may aid in understanding the pathogenesis of posttraumatic OA.	[81]
	A two-sample Mendelian Randomization analysis	Metabolomic studies using plasma/serum have identified altered metabolic profiles in patients with OA, highlighting the potential of metabolomics in understanding the pathophysiology of the disease.	The finding suggested that identified metabolites and metabolic pathways can be considered useful circulating metabolic biomarkers for OA screening and prevention in clinical practice.	[82]
	High performance-liquid chromatography mass spectrometry of synovial fluid from mouse OA model	Investigated the effects of long-term exercise and a high-fat diet on synovial fluid metabolomics and joint structural phenotypes in mice, emphasizing that obesity strengthened synovial fluid metabolite links to blood glucose and inflammation.	These findings provide new insights into the pathogenesis of obesity-related joint diseases and suggest that dietary and exercise interventions may play a significant role in the prevention and management of such conditions.	[84]
	Bioinformatics analysis	Utilized bioinformatics analysis to identify key biomarkers and immune infiltration in the synovial membrane of OA. By constructing a protein-protein interaction network and performing module analysis, they aimed to uncover important factors contributing to OA development.	The hub genes and the difference in immune infiltration in synovial tissue between OA and normal controls might provide new insight for understanding OA development.	[88]
	Liquid chromatography-high resolution mass spectrometry, Human Metabolome Database, authentic standards and/or MS/MS database analysis in urinary	Urinary metabolomics has unveiled shared metabolic pathways and biomarkers between OA and other conditions, such as perturbation in glutamine metabolism, aiding in the enhanced comprehension of OA's pathological mechanisms and offering potential targets for the development of novel therapeutic strategies.	This finding enhances the understanding of OA's pathological mechanisms and identifies shared metabolic pathways, such as glutamine metabolism disruptions, offering potential biomarkers and therapeutic targets for improved diagnosis and treatment.	[91]
	Analysis the data from the Taiwan Biobank Database	The interplay between OA and body mass index has been explored in relation to leptin promoter methylation in Taiwanese adults.	This study indicates that LEP methylation may play a critical role in the progression of obesity-related OA, offering potential clinical insights into understanding the pathological mechanisms of OA.	[95]
	Quantified CpG methylation in human enhancers based on a public dataset and bioinformatics analysis	A genome-wide analysis identifies abnormal DNA methylation patterns in enhancers among patients with knee and hip OA.	The findings indicate that aberrant methylation of enhancers is related to OA phenotypes, and a comprehensive atlas of enhancer methylation is useful for further analysis of the epigenetic regulation of OA and the development of clinical drugs for treatment of OA.	[96]
	Integrated transcriptomic, epigenetic, and functional analysis of OA cartilage using public datasets and in vitro experiments	The methylation status of the CAMP gene promoter has been linked to chondrocyte apoptosis in OA patients, shedding light on the role of epigenetic modifications in OA pathophysiology.	This study offers a new perspective on understanding the mechanisms of disease onset and progression, paving the way for early diagnosis of OA.	[99]
	In vitro experiments validation	The study reveals that matrix stiffness modulates H3K27me3 demethylation by the opening of mitochondrial permeability transition pores and the translocation of plant homeodomain finger protein 8, subsequently affecting the expression of Mmp13 and Bax genes, which exacerbates the progression of OA.	This provides a new perspective to understand the mechanism of OA based on mechanobiology.	[104]
	Single-cell RNA sequencing of immune cell collected from OA mouse	Identified multiple immune cell types in the joint, such as neutrophils, monocytes, macrophages, B cells, T cells, NK cells, and dendritic cells, shedding light on PTOA-associated changes in the immune microenvironment.	This study provides new insight into PTOA-associated changes in the immune microenvironment and highlights macrophage subtypes that may play a role in joint repair after injury.	[115]
	Development of uCoTarget for ultra-high-throughput single-cell joint analysis of multiple epigenetic proteins.	The epigenome plays a crucial role in determining gene transcription and cell fate, as evidenced by the sculpting of the epigenome with histone modifications and transcription factor occupancy.	These technologies enable in-depth analysis of gene expression regulatory mechanisms and cell fate determination processes, offering new insights into the investigation of disease pathogenesis.	[123]
	Single-cell RNA sequencing analysis	This analysis provides valuable information on the cellular biogeography of human bone marrow niches in OA, highlighting joint fold enrichment of all cell type pairs in the given neighborhood.	This spatially resolved, multiomic atlas of human bone marrow provides a reference for investigation of cellular interactions that drive hematopoiesis.	[126]
	16S ribosomal RNA amplicon sequencing of stool samples and multiplex cytokine analysis of blood samples	In a study by Loeser et al., the association of increased serum lipopolysaccharide with obesity-related OA was investigated. While microbial dysbiosis was not found to be associated with OA, the presence of lipopolysaccharide in the blood was linked to obesity-related OA. This suggests a potential mechanism through which gut microbiota may influence the development of OA.	This suggests that gut microbiota may influence the pathogenesis of OA through the action of lipopolysaccharides, providing new insights into the pathological mechanisms of obesity-related OA.	[129]
	16S ribosomal RNA sequencing of stool samples	Wei et al. examined the association between gut microbiota and elevated serum urate levels, suggesting that microbiota dysbiosis may modulate these levels, potentially impacting OA development.	This study suggests that gut microbiota dysbiosis may regulate elevated serum urate levels, potentially influencing the progression of OA. It provides valuable insights into the underlying mechanisms and potential therapeutic strategies for OA.	[131]
	A comprehensive Mendelian randomization study	Conducted a comprehensive Mendelian randomization study to explore the causal link between gut microbiota, neurophysiological states, and bone diseases. Their findings suggest that alterations in gut microbiota can impact cognitive ability, insomnia, and potentially reduce the risk of site-specific fractures and OA.	These findings provide new insights into the prevention and treatment of related diseases.	[134]
	16S ribosomal RNA gene deep sequencing on cartilage samples from knee OA patients and hip OA patients	By analyzing cartilage samples from human knees and hips, it was found that compared to healthy controls, OA samples exhibited reduced microbial diversity, and there was an increase in functions related to lipopolysaccharide production, phosphatidylinositol signaling, and nitrogen metabolism, while the function of sphingolipid metabolism was decreased.	This suggests that the microbiome within OA cartilage may influence the pathogenesis of OA by affecting inflammatory and metabolic processes.	[142]
	Baseline data analysis from the Applied Public-Private Research enabling OsteoArthritis Clinical Headway cohort	Investigated the association of lipid profiles with OA severity in knee and hand joints, suggesting a potential link between lipid composition and disease progression.	It provides new insights into the pathophysiological mechanisms of OA and potential therapeutic targets.	[154]
	Matrix-assisted laser desorption/ionization mass spectrometry imaging and bottom-up proteomics of human cartilages	Investigated the differences in lipidomics and proteomics of cartilage between OA patients with and without type 2 diabetes, it was found that abnormal omega oxidation and fatty acid biosynthesis pathway may lead to imbalance of lipid metabolism.	These findings provide valuable insights into the interplay between OA and diabetes, as well as potential guidance for personalized treatment approaches.	[157]
	Lateral X-ray Analysis of the Left Knee in Osteoarthritis Using MOST Data and Cox Regression Model	Conducted a radiomics analysis of the patellofemoral joint to improve knee replacement risk prediction using data from the Multicenter Osteoarthritis Study. They found that radiomics analysis of the patellofemoral joint could enhance the prediction of knee replacement risk.	The RadScore of the patellofemoral joint on lateral radiographs emerges as an independent prognostic factor for improving knee replacement prognosis prediction. The knee replacement risk score could be instrumental in managing progressive KOA interventions.	[172]
	Integrating MRI radiomics signature of subchondral bone and clinical characteristics	Developed a dynamic nomogram based on MRI-derived radiomics to predict knee pain improvement over two years for OA patients.	The radiomics-based nomogram comprising the MR radiomics signature and clinical variables achieves a favorable predictive efficacy and accuracy in differentiating improvement in knee pain among OA patients.	[173]
	1.5 T MRI scanner analysis of OA patients’ knee	In a radiomics nomogram study based on MRI, researchers developed a predictive model to assess the impact of vitamin D treatment on patients with OA by analyzing radiomic features from MRI images.	This study may contribute to a more accurate assessment of the potential effects of vitamin D treatment, thereby optimizing therapeutic strategies and improving patient outcomes.	[174]

Specifically, based on the content of Table 1, we learn that genomics and transcriptomics have not only identified genetic variations associated with OA but also revealed how these variations influence gene expression and their connection to the disease. Studies in epigenomics and single-cell omics have provided profound insights into the regulation of non-coding genetic information and cellular heterogeneity. Proteomics, metabolomics, and lipidomics techniques further unveil molecular and metabolic changes in the disease, while the applications of microbiomics and radiomics open up new perspectives on microbial interactions and advanced imaging techniques in disease mechanisms and diagnosis. According to the analysis in Table 2, the potential for clinical applications of these omics findings is immense. The combination of genomics and radiomics techniques improves the accuracy of early OA diagnosis, while the development of single-cell and proteomics aids in monitoring disease progression and optimizing treatment strategies. Insights from metabolomics and lipidomics provide a theoretical basis for developing therapeutic drugs targeting specific metabolic pathways, and research in epigenomics and microbiomics may drive new approaches for personalized medicine and prognostic assessment.

Overall, the integrated application of these omics technologies has not only greatly enriched our biological understanding of OA but also significantly promoted the transition towards precision medicine. Future research should continue to explore how to effectively translate these scientific discoveries into clinical diagnostic and therapeutic tools to achieve comprehensive and personalized medical management for OA patients.

Despite the central role that articular cartilage, synovium, synovial fluid, and menisci play in OA research, there are still some limitations in current studies. Firstly, while these research subjects cover almost all the main components of the joint, in-depth studies on the ECM are relatively scarce, despite its key role in maintaining joint health and function. On one hand, the ECM may interact with receptors on immune cells, promoting their function and positioning. On the other hand, changes in the ECM can modulate the function of immune cells [183]. Furthermore, aging alters the mechanical properties of the ECM perceived by the immune system and reduces mechanosensors that downregulate pro-inflammatory pathways, which is closely related to immune regulation during the aging process. However, the complexity of sampling has long limited investigations. The advent of spatial omics has offered novel perspectives and methodologies to address this challenge. Firstly, spatial transcriptomics technology enables the detection of the entire transcriptome within cells while preserving their precise spatial localization. This capability unravels the complex network of cellular communication and matrix changes, which is crucial for understanding the pathogenesis of OA [184]. Secondly, by integrating multi-omics data, spatial omics technologies provide a more comprehensive molecular map, discovering new potential therapeutic targets and biomarkers, which aids in elucidating the molecular mechanisms of disease. High-resolution single-cell and spatial omics technologies further assist researchers in precisely comprehending the molecular characteristics of complex diseases and constructing new molecular atlases [185]. Mass Spectrometry Imaging technology investigates OA at the tissue level, identifying and quantifying proteins while retaining spatial information, which contributes to understanding the molecular mechanisms of OA [186]. Spatial omics technologies can also reveal cellular heterogeneity and microenvironments within tissues, offering better diagnostic and prognostic information and advancing the development of disease-modifying therapies [187]. In summary, spatial omics technologies have significantly propelled research into the pathogenesis of OA and the development of new treatment methods [188].

In addition, studies on bone cells and muscles around the joint are also relatively few, even though they play an indispensable role in the pathogenesis and progression of OA. Secondly, current research often focuses on single tissues or a single omics approach, lacking comprehensive analysis of multi-omics data, which limits our comprehensive understanding of the complex pathological processes of OA [189]. The integration of multi-omics approaches is essential for advancing our understanding of complex diseases such as OA. Multi-omics techniques encompass the synthesis of data from various omics levels, including genomics, transcriptomics, proteomics, metabolomics, and lipidomics, thereby providing a holistic perspective on disease pathogenesis. The MPLEx protocol exemplifies this approach by enabling the simultaneous extraction of metabolites, proteins, and lipids from a single sample, which facilitates comprehensive multi-omics analysis [190]. As demonstrated in the study by Iijima et al., the use of an integrated approach combining meta-analysis and multi-omics data has elucidated the pathogenic mechanisms of age-related KOA in mice [191]. By integrating data from multiple omics levels, including genomics, transcriptomics, proteomics, and metabolomics, the researchers were able to gain a deeper understanding of the molecular pathways involved in OA pathogenesis. This integrated approach highlights the importance of multi-omics techniques in unraveling the complex mechanisms underlying OA. Furthermore, the application of machine learning algorithms to multi-omics data analysis has emerged as a promising avenue for enhancing our understanding of OA [192, 193]. These algorithms can identify patterns and relationships within the complex datasets generated by multi-omics approaches, ultimately leading to the discovery of novel biomarkers and therapeutic targets. In summary, the integration of multi-omics techniques not only ensures the corroboration of effective data but also enhances the accuracy of assessing disease mechanisms and therapeutic efficacy. Moreover, this integrative approach to some extent mitigates the occurrence of false positives in test results, addressing the limitations of single-pathway research that struggles to encapsulate the holistic treatment philosophy of traditional Chinese medicine. The integration of these technologies remains a challenge at present. Future research should focus on developing and optimizing methods that can integrate multiple omics data to gain a more comprehensive understanding of the pathophysiological mechanisms of OA and to facilitate the development of new therapeutic approaches. Moreover, with the advancements in bioinformatics and systems biology, we anticipate the emergence of more tools and platforms that will make the integration of cross-omics data more feasible.

Moreover, the impact of individual differences and environmental factors on OA has not been fully considered, with many studies limited to specific populations and lacking support from global multicenter, large-sample data.

Looking to the future, we anticipate that omics technologies will continue to play a key role in OA research. With the continuous advancement of next-generation sequencing technology and the ongoing improvement of data analysis tools, we will be able to more deeply reveal the molecular mechanisms of OA and the heterogeneity among individuals. This will push us towards more personalized treatment methods, enabling patients to receive more precise treatment plans [194, 195]. At the same time, we also need to pay more attention to the study of the extracellular matrix, bone cells, and muscles around the joint, in order to fully understand the pathological process of OA. Finally, translating the rich data provided by omics technologies into clinical applications requires more research and verification work.

## Data Availability

No datasets were generated or analysed during the current study.

## Abbreviations

OA: Osteoarthritis
DAMPs: Damage-associated molecular patterns
PRRs: Pattern recognition receptors
TLRs: Toll-like receptors
IL-1β: Interleukin-1beta
TNF-α: Tumor necrosis factor alpha
IL-6: Interleukin-6
ROS: Reactive oxygen species
MMPs: Matrix metalloproteinases
GSDMD: Gasdermin D
GWAS: Genome-Wide Association Studies
SNVs: Single nucleotide variants
KOA: Knee osteoarthritis
GRS: Genetic risk scores
PRS: Polygenic risk score
ECM: Extracellular matrix
lncRNAs: Long non-coding RNAs
miRNAs: MicroRNAs
GC-TOF/MS: Gas chromatography - time-of-flight/mass spectrometry
scRNA-seq: Single-cell RNA sequencing
PTOA: Post-traumatic osteoarthritis
CSPCs: Cartilage stem/progenitor cells
RA: Rheumatoid arthritis
MRI: Magnetic resonance imaging
IPFP: Infrapatellar fat pad
iROA: Incident radiographic knee OA
